# Genome-Wide Analyses of Gene Expression during Mouse Endochondral Ossification

**DOI:** 10.1371/journal.pone.0008693

**Published:** 2010-01-13

**Authors:** Claudine G. James, Lee-Anne Stanton, Hanga Agoston, Veronica Ulici, T. Michael Underhill, Frank Beier

**Affiliations:** 1 CIHR Group in Skeletal Development and Remodelling, Department of Physiology and Pharmacology, Schulich School of Medicine and Dentistry, University of Western Ontario, London, Canada; 2 Department of Cellular and Physiological Sciences, University of British Columbia, Vancouver, British Columbia, Canada; Emory University, United States of America

## Abstract

**Background:**

Endochondral ossification is a complex process involving a series of events that are initiated by the establishment of a chondrogenic template and culminate in its replacement through the coordinated activity of osteoblasts, osteoclasts and endothelial cells. Comprehensive analyses of *in vivo* gene expression profiles during these processes are essential to obtain a complete understanding of the regulatory mechanisms involved.

**Methodology/Principal Findings:**

To address these issues, we completed a microarray screen of three zones derived from manually segmented embryonic mouse tibiae. Classification of genes differentially expressed between each respective zone, functional categorization as well as characterization of gene expression patterns, cytogenetic loci, signaling pathways and functional motifs both confirmed reported data and provided novel insights into endochondral ossification. Parallel comparisons of the microdissected tibiae data set with our previously completed micromass culture screen further corroborated the suitability of micromass cultures for modeling gene expression in chondrocyte development. The micromass culture system demonstrated striking similarities to the *in vivo* microdissected tibiae screen; however, the micromass system was unable to accurately distinguish gene expression differences in the hypertrophic and mineralized zones of the tibia.

**Conclusions/Significance:**

These studies allow us to better understand gene expression patterns in the growth plate and endochondral bones and provide an important technical resource for comparison of gene expression in diseased or experimentally-manipulated cartilages. Ultimately, this work will help to define the genomic context in which genes are expressed in long bones and to understand physiological and pathological ossification.

## Introduction

Endochondral ossification (EO) is the process through which the axial and appendicular skeletal elements form via a transient cartilaginous intermediate [1], [2]. The development of temporary cartilage involves the differentiation of mesenchymal precursor cells along the chondrogenic lineage. The different stages of this developmental program occur in a well-defined region, known as the growth plate [3]. In the growth plate, chondrocytes progress through each step of their life cycle in a spatial pattern where cell morphology correlates with the temporal progression of chondrocyte maturation. The region nearest to the end of the bone is the outermost growth plate zone or resting zone. Chondrocytes in this zone appear small and rounded and likely provide the cellular pool for both future articular chondrocytes and chondrocytes undergoing the subsequent stages of growth plate differentiation. The proliferative zone of the growth plate lies adjacent to the resting zone and is populated with actively dividing chondrocytes arranged in characteristic columns of disc-shaped cells. Upon completion of the proliferative period, chondrocytes mature to hypertrophic chondrocytes, which, as their name suggests, are enlarged cells that constitute the hypertrophic zone. When chondrocytes terminally differentiate, they undergo apoptosis, leaving behind a calcified extracellular matrix (ECM) that is remodeled and degraded by invading blood vessels, osteoprogenitor cells and bone-resorbing cells. This coordinated developmental process of chondrocyte proliferation and differentiation is ultimately responsible for longitudinal bone growth and the final length of mature bone.

During early postnatal life, areas of secondary ossification form in the epiphyseal ends of the long bones. Similarly to the process of primary ossification described above, this process consists of chondrocyte hypertrophy, mineralization, vascular invasion and ultimately replacement of cartilage by bone. The growth plate persists between primary and secondary ossification center and continues to direct longitudinal growth of endochondral bones.

Numerous molecular markers characterize the central stages of the chondrocyte life cycle. Chondrogenesis is typified by the expression of Sox transcription factors 5,6 and 9 [1], [4]. Proliferating chondrocytes synthesize an ECM composed mainly of collagen II and aggrecan, among others, while the central ECM molecule expressed in hypertrophic cartilage is collagen X [5]. Factors expressed at the chondro-osseous junction regulate chondrocyte apoptosis and mineralization of the cartilaginous ECM. Late hypertrophic chondrocytes express factors that promote angiogenesis, bone deposition and the secretion of bone-specific cell ECM. These factors include *Vegf* (vascular endothelial growth factor), *Mmp13*(matrix metalloproteinase 13), *Mmp9* and *Ibsp* [6]–[12]. Additional markers of the osteoblast and osteoclast phenotype, including core-binding factor alpha 1/runt-related transcription factor 2 (*Cbfa1*/*Runx2*), acid phosphatase 5, tartrate resistant (*Acp5*) and tumor necrosis factor (ligand) superfamily, member 11 (*Tnfsf11*; RANKL/receptor activator of NF-kappaB ligand) are upregulated in hypertrophic cartilage and cells in the zone of ossification [13], [14].

Generally, gene expression in growth plate chondrocytes is studied by immunohistochemistry, *in situ* hybridization or by isolating cells from various sources including cell lines and primary cell cultures; however, only a few studies have quantified global gene expression in the context of the whole growth plate or as a function of its constituent zones. Studies typically investigate gene expression by using cell lines [15]–[17], isolated early-stage limb mesenchyme [18], whole growth plates [19], or laser-capture microdissection, which requires fixation and decalcification protocols, and sectioning to evaluate gene expression in specific regions of the growth plate [20]–[23]. While all of these approaches have yielded important insights, each suffers from some disadvantages. Currently used *in vitro* approaches have been instrumental in the systematic characterization of both general growth plate trends and the expression of genes in different cell populations of the growth plate, but the physiological context is at times compromised by experimental manipulation. Several studies have investigated the effects of such manipulations on gene expression in chondrocytes and attempted to preserve tissue structure and gene expression [24]–[27]. Cell culture models are, however, limited in their ability to maintain the physiological cell-cell and cell-matrix interactions occurring in the intact growth plate. Similarly, the micromass culture system, which is able to better recapitulate the three-dimensional context of chondrocyte differentiation, suffers from other unfavorable factors such as cellular heterogeneity and the effects of enzymatically disrupting cells and the absence of spatial architecture. The advent of laser capture microdissection permitted the isolation of individual cell populations directly from the growth plate, but can produce spurious results since tissue processing affects tissue integrity and can alter the detection of some expressed genes [24], [28]. In addition, no microarray studies on laser capture microdissection of prenatal mouse cartilage have been described to our knowledge.

Consequently, the objectives of the current study were three-fold: 1. to examine differential gene expression between different zones of embryonic tibiae using manual microdissection; 2. to identify key functional categories associated with individual zones; 3. to evaluate this system in the context of our previously used micromass culture system [29]. This will provide an in-depth assessment of the suitability of three-dimensional cell culture models for temporal and spatial gene expression patterns in cartilage.

## Methods

### Ethics Statement

All animal studies were approved by the Animal Use Subcommittee of the Council of Animal Care at the University at Western Ontario.

### Animals and Manual Growth Plate Microdissection

**Figure 1:**
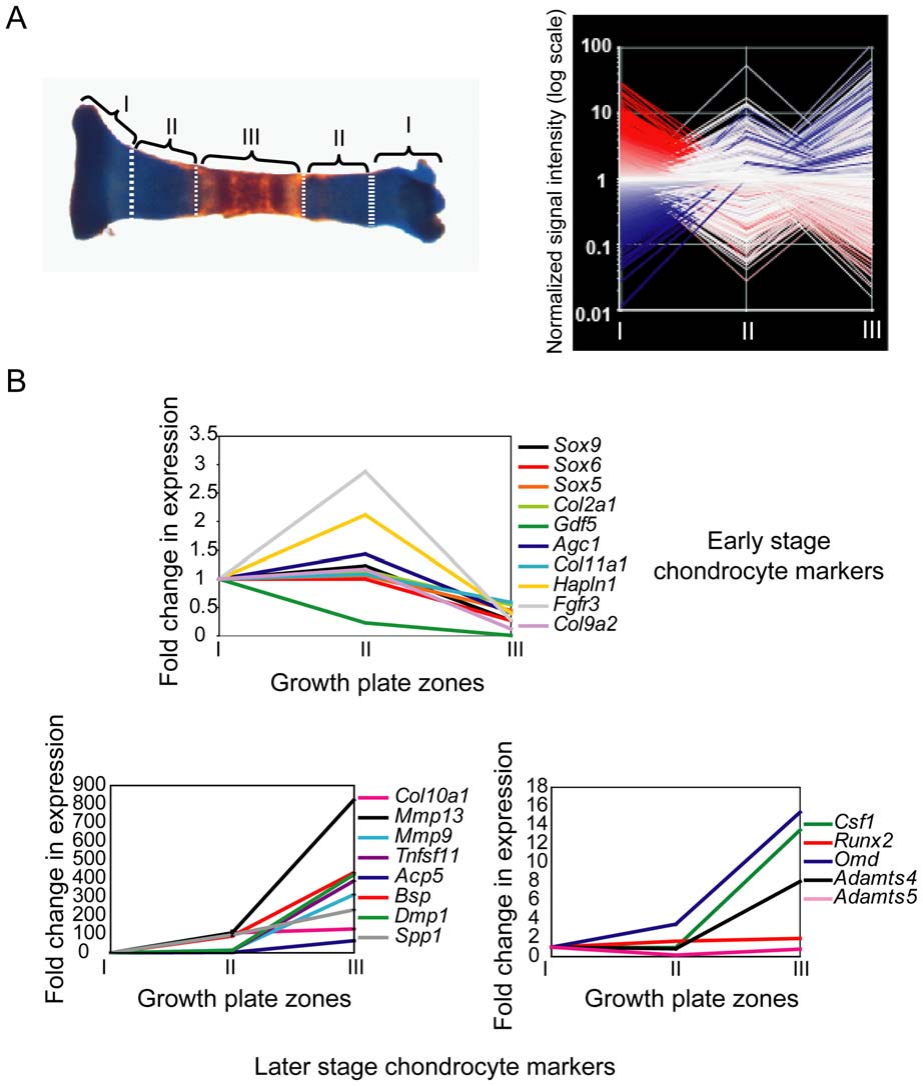
Tibia microdissection and microarray analysis of microdissected zones. Tibiae from 15.5 day old mouse embryos were dissected into three segments called zones I, II and III. Striated lines indicate location of manual segmentation (A, left panel). Growth plate segments for each zone were pooled and total RNA was isolated and hybridized to Affymetrix MOE 430 2.0 chips containing 45 101 probe sets. This experiment was repeated in quadruplicate (A, right panel). Red lines denote probe sets with increased expression in zone I relative to the baseline signal intensity and blue lines denote probe sets that are decreased relative to the baseline signal intensity. White lines represent genes expressed near the baseline intensity. Growth plate microdissection reveals expected expression profiles for established markers of endochondral bone formation. (B) Microarray expression profiles for chondrocyte differentiation sox family members 5, 6 and 9, (*Sox- 5, 6 and 9*), collagen 2 (*Col2a1*), growth differentiation factor 5 (*Gdf5*), aggrecan 1 (*Agc 1*), collagen XI alpha 1 (*Col11a1*), hyaluronan and proteoglycan link protein 1 or cartilage link protein 1(*Hapln1*), fibroblast growth factor receptor 3 (*Fgfr3*) and collagen IX alpha 2 (*col9a2*) are shown (top panel). Late-stage markers of endochondral ossification exhibiting large increases in gene expression in zone III include collagen X (*Col10a1*), matrix metalloproteinase 13 (*Mmp13*), matrix -metalloproteinase 9 (*Mmp9*), tumor necrosis factor (ligand) superfamily, member 11 or receptor activator of NF-kappaB ligand (*Tnfsf11*), acid phosphatase 5, tartrate resistant (*Acp5*), integrin binding sialoprotein (*Ibsp*), dentin matrix protein (*Dmp1*) and osteopontin (*Spp1*) (bottom left panel). Expression profiles for later stage markers that exhibit more moderate increases in zone III included colony stimulating factor 1 (*Csf1*), runt related transcription factor 2 (*Runx2*), osteomodulin (*Omd*), a disintegrin-like and metallopeptidase (reprolysin type) with thrombospondin type 1 motif, 4 and 5 (*Adamts4, Adamts5*) (panel, right panel).

Tibiae derived from four litters of CD1 mice (Charles River) staged at embryonic day 15.5 (E15.5) were isolated and manually separated into three segments based on morphological criteria (Figure 1A, left panel); each litter represented one independent experiment. Starting at either extremity, the region corresponding to approximately the first third of the bone was segmented and designated zone I, which contains both proliferating and resting cells. The next zone corresponding to the second third of the bone was named Zone II and contains mostly prehypertrophic and hypertrophic chondrocytes. The final zone, zone III, constitutes both the most mature hypertrophic chondrocytes and the mineralized portion of the tibiae.

### RNA Isolation, Microarray Analysis and Quantitative Real-Time Polymerase Chain Reaction

Growth plate segments from zone I, II and III were pooled for all animals within one litter. Total RNA was immediately isolated using Qiazol lysis reagent and RNEasy Mini Kit columns (Qiagen) according to the manufacturer's protocol as described [30], [31] RNA quantity and integrity was assessed using the RiboGreen RNA Quantitation Kit (Molecular Probes), and samples were processed and hybridized to the Affymetrix MOE430 2.0 mouse genome chips at the London Regional Genomics Facility. All data is MIAME compliant and the raw data has been deposited in a MIAME compliant database (GEO). The microdissected microarray data set is archived in the gene expression omnibus repository (GEO accession: GSE7685). Quantitative real-time polymerase chain reaction (qRT-PCR) amplification was completed using the ABI Prism 7900 Sequence Detection System (Applied Biosystems). Triplicate reactions were executed for each sample of each of four independent trials. TaqMan one-step master mix kit (Applied Biosystems) and gene-specific target primers and probes were used for amplification. TaqMan GAPDH control reagents for house-keeping gene Glyceraldehyde-3-phosphate dehydrogenase (*Gapdh*, forward primer 5′-GAAGGTGAAGGTCGGAGTC; reverse primer 5′-GAAGATGGTGATGGGATTTC; probe JOE-CAAGCTTCCCGTTCTCAGCC-TAMRA) were used as an internal amplification control. Integrin-binding sialoprotein *(Ibsp)*, dentin matrix protein (*Dmp1*), cyclin-dependent kinase 1c, (*Cdkn1c*), *Col2a1*, Indian hedgehog (*Ihh)* and *Sox9* were assayed using the TaqMan® gene expression assays in accordance with the manufacturer's directions. Amplified transcripts were quantified using the standard curve method, and the relative transcript abundance was determined by calculating the quotient of the gene of interest and equivalent *Gapdh* values. Fold changes in expression were calculated relative to zone I. Statistical significance was determined by one-way ANOVA analysis and a Bonferroni's multiple comparison Test. P-values less than 0.05 were deemed significant.

### Data Analysis

Microarray data was pre-processed using the GC (guanine and cytosine) Robust Multi-chip Averaging (RMA) algorithm in Genespring GX*. Expression values were further filtered by retaining only those probe sets with expression values of at least 50 in at least 25% of all conditions, thus generating a list of 22 497 probe sets. Subsequent zone comparisons from the microdissected tibiae data set (MD) were filtered using a 1.5-fold change threshold that produced lists of 6185, 8134 and 7220 probe sets for the zone I vs. II, II vs. III and zone I vs. III, respectively (Table 1).

**Table 1:** Microarray analysis of microdissected embryonic growth plates.

Specifications	Probe sets I vs. II	Probe sets II vs. III	Probe sets I vs. III
Total number of probe sets	45101	45101	45101
Significantly expressed	22497	22497	22497
1.5 fold change	6185	8134	7220
5 fold change	474	937	766
10 fold change	170	366	288
1.5 fold upregulated	3433	4005	3678
5 fold upregulated	317	262	305
10 fold upregulated	102	87	115
1.5 fold downregulated	2752	4129	3542
5 fold downregulated	157	675	461
10 fold downregulated	68	279	173

The same data set was normalized in parallel using RMA using RMAEXPRESS software v.0.4.1 developed by B. Bolstad, University of California, Berkeley [32]. Background adjustment and quantile normalization parameters were selected for data processing. Logarithmically transformed expression values were used to implement Gene Set Enrichment Analysis (GSEA).

The micromass culture data set (MM) [29] (GEO accession: GSE2154) was similarly processed for comparative studies. After discarding poorly expressed probe sets, a list of 13 185 probe sets was obtained. Additionally, analogous comparisons involved day 3 vs. 9, day 9 vs. 15 and day 3 vs. 15 lists which contained 3828, 2005 and 5580 probe sets, respectively, upon being filtered using a 1.5-fold change filter (Table 2).

**Table 2:** Comparisons between microdissected and micromass arrays.

Classifications	Probe set number
Total number of probe sets	45101
Probe sets expressed in MD*	22497
Probe set expressed in MM**	13185
Probe sets common to both lists	11806
Probe sets only found in MM	1379
Probe sets only found in MD	10691
Probe sets in 3 vs. 9	3828
Probe sets in 9 vs. 15	2005
Probe set in 3 vs. 5	5580
Probe sets found in 3 vs. 9 AND I vs. II	1323
Probe sets found only in 3 vs. 9	2505
Probe sets found only in I vs. II	4862
Probe sets found in 9 vs. 15 AND II vs. III	886
Probe sets found only in 9 vs. 15	1119
Probe sets found only in II vs. III	7248
Probe sets found in 3 vs. 15 AND I vs. III	2037
Probe sets found only in 3 vs. 15	3543
Probe sets found only in I vs. III	5183

note: fold changes include genes that are both up and downregulated.

* microdissected tibiae data set.

** micromass culture data set.

### Gene Ontology Annotations

Probe set lists composed of I vs. II, II vs. III and I vs. III zone comparisons exhibiting at least 1.5-fold changes in gene expression between each respective condition were used as inputs for Genespring GX Gene Ontology annotations.

### Gene Set Enrichment Analysis (GSEA)

The GSEA algorithm was implemented with GSEA v2.0 software [33], [34]. Ranked expression lists were derived from RMAEXPRESS and GeneSpring GX® 7.3.1. Briefly, the GSEA algorithm ranks all array genes according to their expression under each experimental condition. The resulting ranked metric score (RMS) is therefore a function of the correlation between a gene's signal intensity, the experimental conditions in question and all other genes in the data set. Enrichment score (ES) are then calculated for *a priori* gene lists or gene sets that are associated with a particular molecular classification. In our analysis, gene sets were created from different functional groupings, molecular classifications, tissues, and other microarray screens [35]. Ranked enrichment scores (RES) that determine the extent to which individual genes from a gene set are represented at the extremes of the ranked gene list are then calculated. Specifically, these values are obtained by walking along the ranked list using a cumulative sum statistic which increases when a member of a particular gene set is found in the ranked gene list and zone and is coordinately penalized when it does not appear in the gene set. A null distribution of ES is subsequently generated by permutation filtering to evaluate the statistical significance of the observed RES values. Permutation filtering randomly assigns the experimental conditions or class labels (i.e., I vs. II) to the different microarray samples. After this procedure has been repeated for each gene set, the ES are normalized (NES) to account for differences in gene set size. The false discovery rate (FDR) is then calculated relative to the NES values to determine the false-positive rate. Significant FDR and p-values were defined as less than 25% and 0.001, respectively, in accordance with GSEA recommendations.

### Gene Set Creation

#### User defined gene sets

Gene sets were generated using the probe set search tool and the molecular function class of Gene Ontology annotations in GeneSpring GX. Additional gene sets were created using lists from pair-wise comparisons between day 3 and 9, day 9 and 15 and day 3 and 15 of a previously generated micromass data set ([29], Table 3). A total of 3828, 2005, 5183 probe sets showing a minimum 1.5-fold change in gene expression were used for the 3 vs. 9, 9 vs. 15 and 3 vs. 15 lists. Probe set redundancy was eliminated in all gene sets using the CollapseDataset function in the GSEA program. All probe set identifiers were converted to the Human Genome Organization (HUGO) annotations. Probe sets lacking corresponding HUGO annotations were excluded from the analysis. Default parameters were used to execute the analysis and median values taken to represent the range of duplicated probe sets for a given gene. A total of 90 user-defined gene sets were generated from GeneSpring-derived annotations for various molecular classifications.

**Table 3:** Gene sets used in GSEA.

Category name	Number of genes	Category name	Number of genes
Adipose	70	Fkbp	33
Apoptosis	39	Igf	48
Bone	116	Cart 2	299
Cartilage	28	Cart 3	352
Catalytic	245	Liver 1	260
Chaperone	81	Liver 2	260
Chemokine	31	Blood	111
Chromatin Hdac	24	Protease 1	269
Cyclin	225	Protease 2	269
Cytokine	127	Phosphatase	473
1 Dnabind	500	Dusp	20
2 Dnabind	448	Kinase 1	499
Ecm	228	Kinase 2	499
Electron Transp	40	Kinase 3	227
Gf Receptor	327	Integrin Rel	173
Gluconeogen	31	Brain Rel	379
Growth Factor	106	Hepatocyte	19
Gtpase Activator	46	Obl Oclast	16
Gtpase Activ	73	Interleukinrelated	175
Heparin Bind	37	Rgs Related	44
Hormone	75	Caspase Related	47
Muscle	198	Creb Atf3	32
Neg Apoptosis	50	Nuclear Receptor	138
Oncogene	154	Nuc Hormone Receptor	55
Pos Apoptosis	79	Pzhorton.Farnum	413
Related Apoptosis	311	Hzhorton.Farnum	407
Structure	151	3 vs. 9 1	495
Sugar Bind	104	3 vs. 9 2	495
Tf Activ	56	3 vs. 9 3	495
Tf Repress	55	3 vs. 9 4	495
Tgfb	45	3 vs. 9 5	495
Tnf Receptor	69	3 vs. 9 6	276
Tumor Suppressor	48	9 vs. 15 1	497
Wnt	53	9 vs. 15 2	497
Actin Cytoskel	38	9 vs.15 3	497
Angiogen	57	9 vs. 15 4	39
Bmprelated	62	3 vs. 15 1	497
Cytoplasm	411	3 vs. 15 2	497
Erk Related	40	3 vs. 15 3	497
Fgf Related	64	3 vs. 15 4	497
Membrane	260	3 vs. 15 6	497
Mapkrelated	267	3 vs. 15 5	497
Metabolism	196	3 vs. 15 7	497
Nucleus 1	494	3 vs. 15 8	462
Nucleus 2	494		
Nucleus 3	510		

#### Gene sets from the Molecular Signature Database

To provide an unbiased assessment of the similarities between the micromass (MM) and microdissected (MD) data sets, enrichment of specific cytogenetic loci, molecular pathways and regulatory motifs in different zones of the growth plate, we used the GSEA algorithm in combination with gene sets available from the GSEA Molecular Signature Database (MgSigDB) (http://www.broad.mit.edu/cancer/software/gsea/msigdb/msigdb_index.html). The c1 = chromosomal location, c2 = functional, c3 = motifs gene sets were used. Specifically, the c1 is composed of 325 gene sets containing information about the cytogenetic locations of HUGO and Unigene annotated genes. The c2 data set is a curated, 1137 gene set matrix containing information about specific biological, metabolic and signaling pathways as gene ontologies, chemical and genetic perturbations, disease phenotypes and animal models. Microarray screens published in the biomedical literature were additionally included in the c2 gene set. The c3 matrix contained 173 gene sets containing functional motifs conserved in the human, mouse, rat and dog genomes including putative transcription factor binding sites from TRANSFAC, a transcription factor binding site search program and data base, candidate motifs and microRNA target sequences. Results with a p<0.001 and FDR<0.25 are shown.

## Results and Discussion

### Transcriptional Profiling of Embryonic Growth Plate Zones and Data Validation

The embryonic tibia is composed of several morphologically distinct zones that mirror the different phases of the chondrocyte life cycle. To elucidate the correlation between morphological differences in growth plate regions and their corresponding gene expression patterns, we isolated tibiae from E15.5 day old mouse embryos. Tibiae were manually divided into three zones (Figure 1A, left panel). RNA samples from each zone were subsequently isolated and hybridized to Affymetrix MOE 430 2.0 arrays for microarray analysis. Upon completion of all data processing, we obtained a list of 22 497 probe sets expressed in at least one zone (Table 1, Figure 1A, right panel).

To confirm the expression of established markers of endochondral ossification found in our array to the literature, we first examined our microarray data sets for the expression of early stage chondrocyte markers such as Sox family members 5,6 and 9 (*Sox5,6* and *9*), *Col2a1*, growth differentiation factor 5 (*Gdf5*), *Agc 1*, *Col11a1*, *Hapln1*, *Fgfr3* and *Col9a2* (Figure 1B, top panel). Overall, these markers exhibited expected and consistent expression patterns in that higher expression occurred in zone I and lower expression occurred in zone III. Zone II markers showed less consistency in both the amplitude of the signal intensity and the actual pattern of gene expression [4], [36]–[40].

Expression of late stage chondrocyte markers such as *Col10a1* and molecules involved in matrix turnover, EO, and osteoblast and osteoclast differentiation were consistent with their expected expression patterns [8], [12]–[14], [19], [41]–[51]. Specifically, *Mmp13*, *Mmp9*, *Tnfsf11*, *Acp5*, *Ibsp*, *Dmp1*, osteopontin (*Spp1/Opn1*), colony stimulating factor 1 (*Csf1*), *Cbfa1*/*Runx2*, osteomodulin (*Omd*), a disintegrin-like and metallopeptidase (reprolysin-type) with thrombospondin type 1 motif, -4 (*Adamts4*) and -5 (*Adamts5*) were all up-regulated in zone III (Figure 1B, bottom). These data are consistent with zone I corresponding to the resting and proliferating chondrocytes, zone II consisting mostly of pre-hypertrophic and early hypertrophic chondrocytes, and zone III consisting of late hypertrophic chondrocytes and possibly small populations of invading osteoblasts, endothelial cells and osteoclasts.

**Figure 2:**
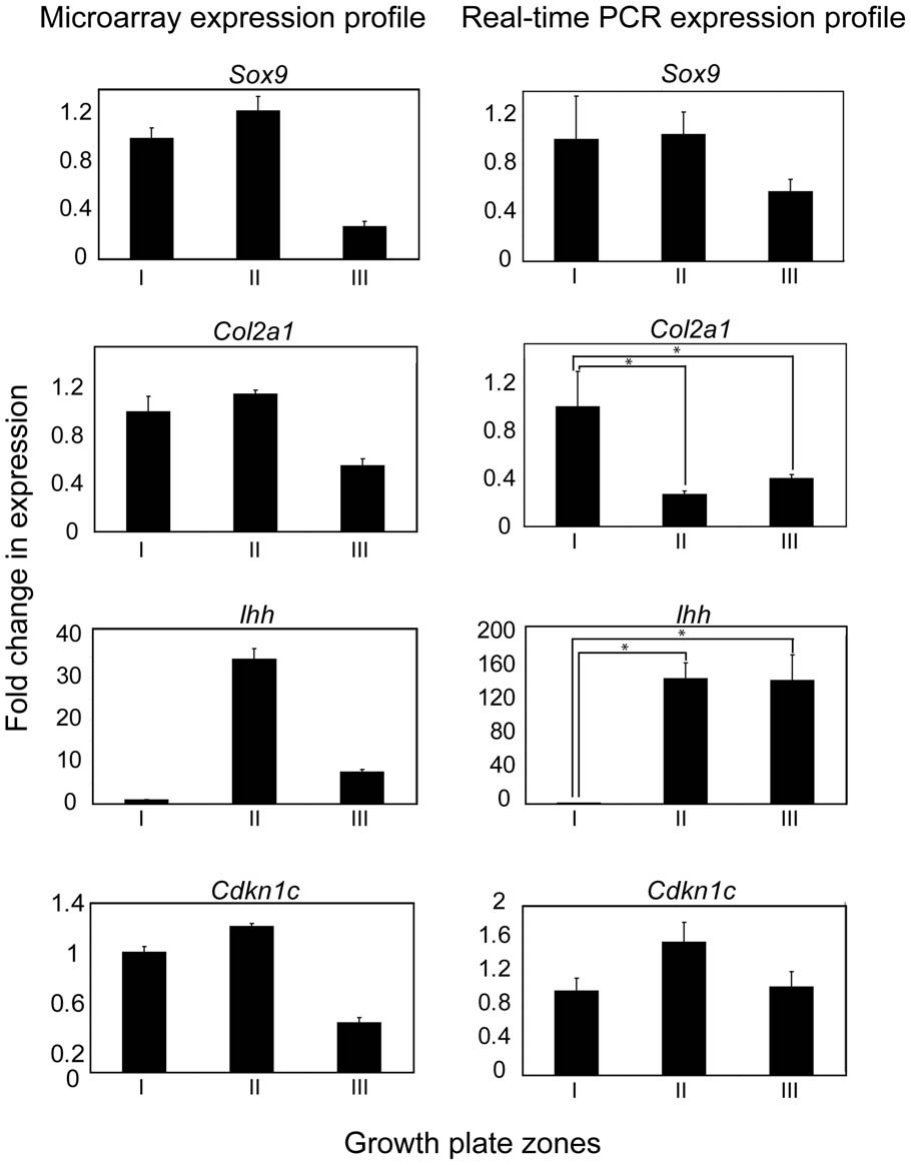
Real-time RT-PCR confirmation of early-stage markers of chondrocyte differentiation. Expression profiles of known chondrocyte markers were evaluated with qRT-PCR. Zone-specific expression of SRY (sex determining region Y)-box 9 (*Sox9*), collagen 2 (*Col2a1*), Indian hedgehog (*Ihh*) and cyclin-dependent kinase inhibitor 1c (*Cdkn1c, encoding p57*) profiles are shown on the right, with the corresponding microarray data on the left. Four independent RNA isolations were evaluated for each probe and primer pair and p-values less than 0.05 were deemed significant.

In addition to confirming the expression of known markers by comparing our microarray data to previously documented findings, we verified transcript accumulation of selected factors with qRT-PCR. We selected several known growth plate markers including *Sox9*, *Col2a1*, *Ihh* and *Cdkn1c* (encoding the cell cycle inhibitor p57). In accordance with the literature, *Sox9* and *Col2a1* expression was highest in zone I and zone II with lower expression in zone III, which corresponds to the cartilage-bone interface [52], [53] (Figure 2). The correlation between microarray gene expression profiles and qRT-PCR for these two markers was not, however, exact, as we noticed decreased *col2a1* expression in zone II compared to Zone I in the qRT-PCR analysis (which is the expected trend), while col2a1 expression was similar between these 2 zones in microarray data. This discrepancy warrants the use of alternate techniques in addition to microarray analysis to precisely quantify transcript and protein levels in each zone. In addition, expression profiles for *Ihh* and *Cdkn1c* deviated from expected patterns. In the case of *Ihh*, the microarray expression profile demonstrated highest expression in zone II with lower expression patterns in both zones I and III. Conversely, qRT-PCR demonstrated that *Ihh* expression is maintained at higher levels in zone III. Alternate confirmation with *in situ* hybridization and immunohistochemistry is required to localize Ihh expression. These results indicate that microarray data for highly expressed markers are consistent with the literature; however, genes with lower gene expression levels exhibit greater variability. *Cdkn1c* is expressed in post-mitotic cells of the growth plate [3], [54], [55] but we did not find the anticipated trends between the zones. We were able to establish differential *Cdkn1c* (p57) expression in zones I and II of the growth plate with qRT-PCR (Figure 2) and immunostaining of embryonic growth plates for p57 protein [56]. The presence of p57 immunostain in articular chondrocytes [56] could account for the signal obtained for the resting/proliferating zone.

**Figure 3:**
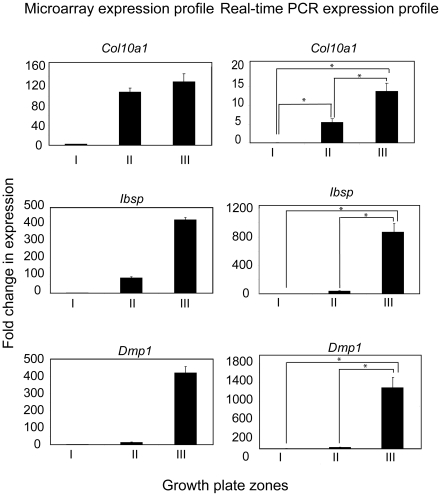
Real-time RT-PCR confirmation of later-stage markers of endochondral ossification. RNA samples from microdissected embryonic tibiae were used to confirm microarray expression profiles for later-stage chondrocyte markers. Collagen X (*Col10a1*), bone sialoprotein (*Ibsp*) and dentin matrix protein 1 (*Dmp1*) were identified in the microdissected array and validated with qRT-PCR. Four independent RNA isolations were evaluated for each probe and primer pair and p-values less than 0.05 were deemed significant.

In parallel, we evaluated the expression patterns of late stage ECM molecules including *Col10a1*, *Ibsp* and *Dmp1* by qRT-PCR (Figure 3). *Ibsp* and *Dmp1* were expressed similarly in both assays in which they exhibited increases in the range of 100- to 1000-fold in zone III of the tibia. *Col10a1* was an exception in that it exhibited a lower fold change difference using qRT-PCR compared to the microarray data. We expected a certain degree of variability between both methods, given the limitations of the normalization algorithms used prior to assessing fold change differences [57]. Overall however, the trends of these results are consistent with the time line for matrix turnover in EO in that remodeling of the chondrocyte ECM follows hypertrophic differentiation [58], [59]. Therefore, evaluation of gene expression patterns of prototypical chondrocyte markers within different zones with qRT-PCR serves as proof of concept for the utility of this system for studying relative gene expression patterns.

### Functional Annotations of Microdissected Growth Plate Gene Expression Patterns

**Figure 4:**
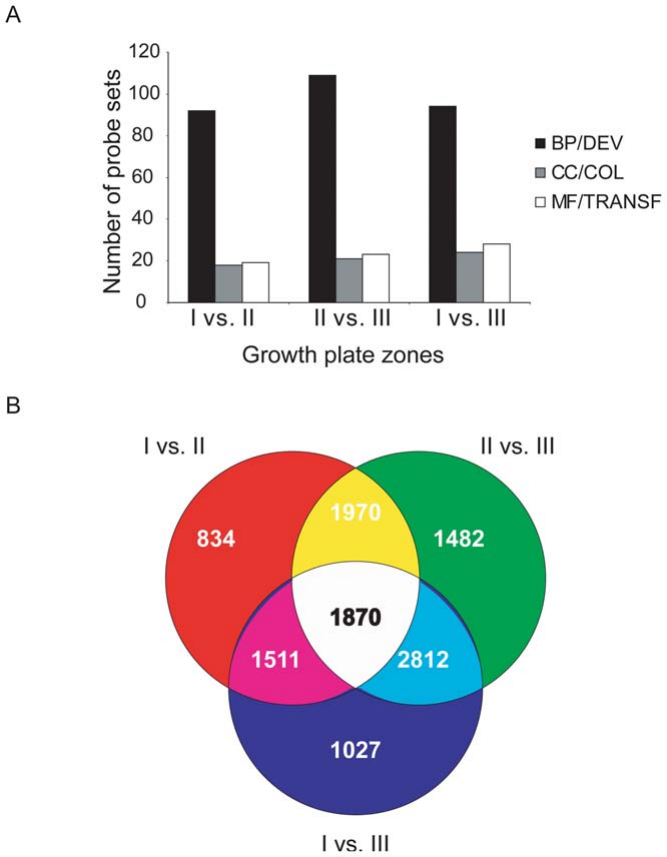
Gene ontology annotations of microdissected growth plate zone comparisons. Lists of probe sets subject to pair-wise comparisons between zones I and zone II (I vs. II), zone II and zone III (II vs. III) and zone I and zone III (I vs. III) were each classified according to biological process (BP), cellular component (CC), and molecular function (MF) (A). The most significant hierarchy was followed in each case until the smallest significant sub-classification was found. In each list, developmental (DEV), collagen (COL) and transporter functions (TRANSF) were identified. P-values less than 0.001 were deemed significant in each case. The number of probe sets both common to each respective zone comparison and unique to a given list was illustrated using a Venn diagram (B).

To classify pervasive functional categories in endochondral ossification, we created lists of pair-wise comparisons between the different zones using a 1.5-fold change filter. The resulting lists contained 6185, 8134 and 7220 probe sets for zone I vs. II (I vs. II), zone II vs. III (II vs. III) and zone I vs. III (I vs. III) comparisons, respectively (Table 1). We then overlapped these lists with the biological process (BP), cellular component (CC) and molecular function (MF) categories in the Gene Ontology browser of GenespringGX (Figure 4A). Approximately 52% of all genes contained in these lists have corresponding Gene Ontology annotations. In each zone comparison, “development”, “collagenous” probe sets and “molecular transport” were deemed the most significant categories associated with BP, CC, and MF, respectively (Figure 4A). Although the same general BP and CC categories were identified in each zone comparison, the probe sets making up these categories were not identical in each comparison and only overlapped by 50%. The MF category did not contain any similar probe sets. Therefore the categories of development, collagen and transport genes were similarly prominent in lists of genes changing among all growth plate zones, but the specific genes belonging to these gene lists were not the same in the different comparisons.

The identification of similar molecular categories irrespective of the zone comparison evaluated emphasizes the concept of networks and functional categories being responsible for regulating the chondrocyte phenotype, rather than just individual genes. We next endeavored to eliminate genes that exhibited changes among all growth plate zones from our subsequent studies. We identified 1870 probe sets in zone intersections between all three pair-wise comparisons, 2812 probe sets common to zones I vs. III and zone II vs. III lists, 1511 probe sets common to the I vs. II and I vs. III comparisons, and 1970 common to the I vs. II and II vs. III lists (Figure 4B). We focused on classifying the 834, 1482, and 1027 probe sets that were unique to I vs. II, II vs. III and I vs. III comparisons, respectively. For example, in the BP category, probe sets involved in DNA replication, protein biosynthesis and gene silencing were most significant in I vs. II, II vs. III and I vs. III zone comparisons, respectively.. It is therefore likely that the morphological differences in the embryonic tibia are mirrored, to some extent, by zone-specific gene expression patterns; for example, genes involved in protein synthesis exhibit larger changes in the zone II vs. III comparison where cells undergo hypertrophic cell growth.

### Gene Expression Patterns in Growth Plate Zones

Our next objective was to identify molecular classes that were enriched in the different growth plate zones by GSEA. GSEA provides a means to describe how a gene list (e.g. the genes from a zone I to II comparison) correlates positively or negatively with other preset gene sets, for example gene sets corresponding to tissue-specific gene expression or specific functional categories [34]. We compared the expression of genes found in zone I to genes expressed in zones II and III, and finally between zone II and zone III. We then compared these lists to a matrix of 90 user-defined gene sets (Table 3) belonging to various molecular classifications (see methods for details). Significantly enriched classes had p-values less than 0.001 and maximum FDR of 25%. Gene sets corresponding to ECM, growth factors, angiogenic factors and chemokines were all enriched in zone I when compared to zone II (Table S1-1–9, see supplemental data). Gene sets were also made from analogous comparisons between different stages of the chondrocyte development in micromass cultures (MM) [29]. These gene sets were enriched in our analysis, which supported the suitability of primary cell culture models in studying gene expression in chondrocytes. Enrichment of the user-defined gene sets in zone II, however, was limited since the only significant gene set contained genes associated with chaperone activity (Table S1–6, see supplemental data). Zone II gene sets that met the FDR cutoff, but not the p-value cutoff, included phosphatases, metabolic molecules, genes involved in TGFβ signaling and bone markers.

Comparisons between zones II and III revealed a similar trend in which gene sets in zone II did not fulfill all criteria for significant enrichment, while true enrichment in zone III was more extensive (Table S2–1, see supplemental data). Cartilage genes, molecular chaperones, metabolic genes and genes involved in gluconeogenesis were enriched in zone II (Table S2–2–5,-9, see supplemental data). In zone III, genes sets containing molecules involved in heparin binding, angiogenesis and chemokines, among others, were enriched (Table S2–6–8, see supplemental data). Comparisons between zones I and III revealed no enrichment in zone I (when considering the p-value threshold), but extensive enrichment in zone III with gene sets for blood, phosphatases, cartilage and MAPK (Table S3–1–10, see supplemental data).

These results provided further evidence that genes regulating endochdondral ossification present a combination of zone-specific genes and broadly expressed genes. Cartilage gene sets were significantly enriched in all zones; however, only the II vs. III and I vs. III categories contained similar genes including *Sox9*, *Hapln1* and *Agc1* (Table S2–9, S3–11, see supplemental data). The cartilage genes enriched in zone III vs. I contained a different subset of transcripts including *Dmp1* and *Mmp13*, which we had identified in a previous study [29] and/or confirmed with qRT-PCR analysis. Bone transcripts were enriched in zone II and zone III, respectively, when compared to zone I. Core genes found in these groups included *Bmp7*, parathyroid hormone receptor 1 (*Pthr1)*, *Tnfsf11*, and *Ibsp*, which are prototypical markers of EO (Table S1–7, S3–12, see supplemental data) [60].

While many of the expected functional classifications and markers were expressed, genes used to calculate the enrichment scores of the significantly enriched gene sets were not necessarily known cartilage markers. It is therefore likely that the differences we observe in the different growth plate regions could be a function of network connections rather than only its individual constituents. This reinforces the concept that key zone-specific regulatory molecules are supported by the expression of numerous other molecular players. It is interesting to note that the cartilage gene set taken from another published microarray screen failed to be significantly enriched in our growth plate comparisons [23]. This observation likely highlights the effect of experimental methods on gene expression. In addition to species (mouse vs. rat) and age (i.e. fetal vs. adolescent) differences, the actual processing of the cells used for gene expression analysis differed. In our study, total RNA was directly isolated from tibiae while Wang's study, which used laser-capture microdissection of rat growth plates, involved cryogenic sectioning before expression analysis [23].

These analyses suggest that zones I and III show well defined enrichment patterns and consequently zone-specific expression. Gene expression in zone II seems less distinct, which is consistent with the transitions chondrocytes must undergo to presage bone deposition. Additional studies that address the precise gene expression differences in the transition regions between zones I vs. II and II vs. III of the tibia are necessary to more accurately define the markers expressed as chondrocytes terminally differentiate.

### Comparisons between Micromass and Microdissected Data Sets

Next, we wanted to determine whether the genes expressed in the microdissected embryonic tibiae were comparable to our previously used micromass (MM) culture system [29]. Based on established knowledge of gene expression in the MM system, we matched pair-wise comparisons between the three microdissected (MD) growth plate zones to pair-wise comparisons between different MM culture days. Only those comparisons that demonstrated the largest similarity between both experiments were selected for further analysis. Differential expression between days 3 and 9 of MM culture was likened to zone I vs. II comparison; day 9 vs. 15 of MM culture was analogous to zone II vs. III comparison and day 3 vs. 15 of MM culture was compared to zone I vs. III. The first phase of our analysis involved determining whether genes differentially expressed in the MD data set were similarly expressed in the MM data set.

**Figure 5:**
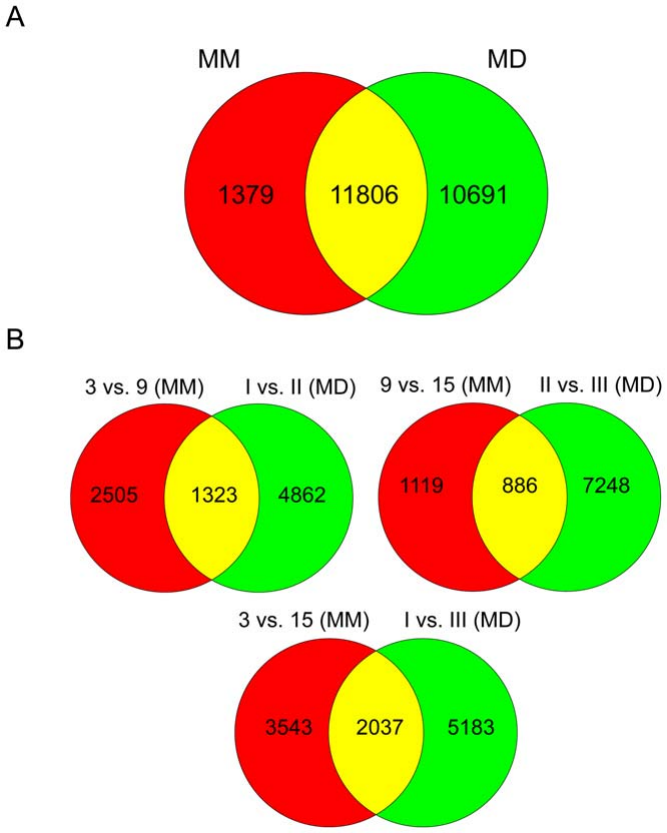
Genes expressed in both microdissected embryonic tibiae and primary mesenchymal micromass cultures. Venn diagrams delineating the overlap between probe sets expressed in the microdissected (MD) tibiae array data set and the micromass (MM) array data set (A). Pair-wise comparisons between individual growth plate zones are compared to their similar pair-wise comparisons in the micromass culture data (B). Specifically, probe sets differentially expressed between days 3 and 9 (3 vs. 9) of micromass culture are compared to the I vs. II list. Similarly, probe sets differentially expressed between days 9 and 15 (9 vs. 15) and days 3 and 15 (3 vs. 15) of micromass culture are compared to probes identified in II vs. III and zone I vs. III lists, respectively.

We first compared the number of probe sets significantly expressed in either the MM or MD data sets and found 11 806 common probe sets, 1 379 probe sets found only in the MM data set and 10 691 probe sets found only in the MD data set (Figure 5A). Since we were comparing probe sets from two different array platforms, namely the MOE 430A (MM) and MOE 430 2.0 (MD) chips, the former of which contains half the probe sets of the latter, we anticipated and confirmed that approximately 50% of probe sets from the MD data would match corresponding probes in the MM data set. Also, it is likely that day 3 of MM culture represents a slightly earlier stage of chondrocyte development compared to MD zone I. The discrepancy between day 3 of MM and zone I of the growth plate could additionally be due to the fact that MM are derived from the mesenchyme which has the potential to develop into numerous cell types (of myogenic, adipogenic and osteogenic lineages) and is therefore a heterogeneous cell population, whereas zone I is mostly constituted of chondrocytes.

The next phase of the analysis involved comparing our MM time point comparisons to our MD zone comparisons (Figure 5B). In this case, approximately 40% of transcripts were common between MM and MD data set using the MM values and 20% when using the MD numbers. However, it is apparent that the majority of the genes identified in the pair-wise comparisons were not shared between MM and MD.

**Figure 6:**
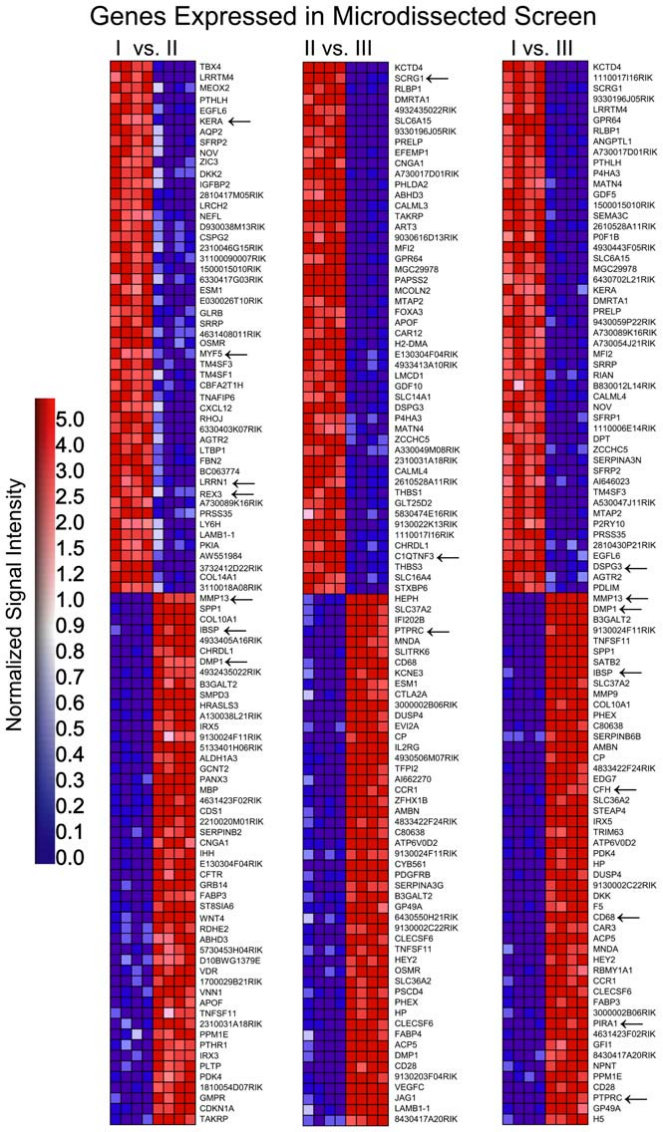
Similarities between the micromass culture time course and microdissected growth plate data sets. Heat maps of genes exhibiting highest differential expression and positive correlations to either zones I or II in the pair-wise microdissection comparisons are shown. Signal intensities are illustrated by varying shades of red (up-regulation) and blue (down-regulation). Arrows indicate genes common to the corresponding micromass comparisons.

**Figure 7:**
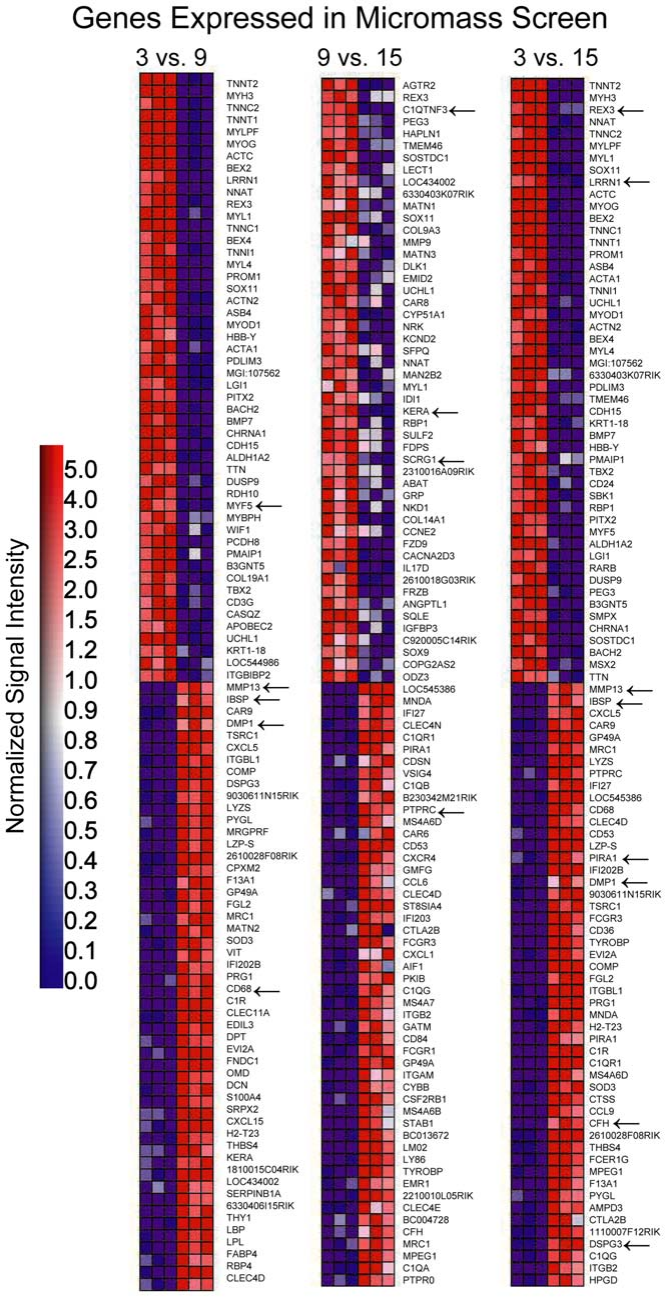
Heat map of micromass culture data set analyzed by GSEA. GSEA-derived heat maps of the top 100 differentially expressed probe sets enriched in micromass data. Correlations between probe sets and day 3 or day 9 of micromass culture for the first map, day 9 or day 15 for the second map and day 3 or day 15 for the third map are shown. Expression profiles for all experimental replicates are depicted for each time point. Signal intensities are illustrated by varying shades of red (up-regulation) and blue (down-regulation). Arrows indicate genes common to the corresponding microdissected growth plate zone comparisons.

Therefore, we asked whether the most significantly changed genes were common between the MM and MD data. Only 15 of the 100 most significantly changed genes were similar between the experimental systems (Figure 6 and 7).

We postulated that these results could reflect the limitations of inter-array comparisons and nuances in the way each experiment was executed. However, our previous analysis (in which gene lists derived from the MM array were enriched in the MD data set) suggests that these comparisons did not reflect the full extent of the similarities between both data sets. These analyses did, however, point to the fact that on the level of individual genes, with the exception of prototypical chondrocyte markers, our MM culture days could be different from our tibia zones. We aimed to further corroborate these findings by analyzing both data sets by GSEA in parallel. We used a series of gene sets from the Molecular Signature Database that contains genes grouped based on chromosomal region (c1), pathways (c2) and common motifs (c3). In cases where the interrogated gene list had a FDR that was above the 25% cutoff, we selected the top 20 gene sets for comparison.

### c1 Comparisons

To obtain an objective view of the similarities and differences between the MM and MD data sets, we decided to look at several molecular categories. The first was cytogenetic loci, corresponding to the c1 gene set matrix from MgSigDB. Specifically, we compared the NES scores assigned to each cytogenetic locus that was correlated with a particular time point or growth plate zone comparison. We compared MM day 3 vs. 9 to MD zone I vs. II data and identified 6 enriched cytogenetic locations (Table S4–1,2, see supplemental data). In all cases except for one (Table S4–1,2), days 3 vs. 9 and zones I vs. II were similarly enriched.

Another emerging pattern included enrichment in day 9 and zone II (Table S4–3,4) and correlated with loci associated with skeleton-related chromosome mutations and genetic determinants related to the bone phenotype [61]–[65]. The 9 vs. 15, II vs. III comparisons show a total of 10 enriched gene sets, all but one of which showed the expected similar enrichment pattern (Table S4–3,4, see supplemental data). Other loci enriched at day 9 and zone II or day 15 and zone III were similar in their association with bone-related loci [66]–[71]. Four main enrichment patterns occurred in the day 3 vs. 15 and zone I vs. III comparison (Table S4–6). Two chromosomal gene sets,deviated from the expected trend in the day 3 vs. 15 and zone I vs. III comparison (Table S4–6). This pattern appeared to be counter-intuitive since day 3 and zone III represent two opposite stages in the chondrocyte life cycle, but it could be explained by the fact that both loci are linked to the skeleton. [72], [73]. In addition, three loci exhibited common enrichment in day 3 and zone I, and the remaining seven gene sets were enriched in day 15 and zone III. These gene sets were also linked to the skeleton [49], [74]–[76]. Therefore, in most cases, enrichment patterns for different cytogenetic loci are conserved; however, the relationship between the identified loci and the individual genes making up the probe sets belonging to MM and MD data sets are not congruent.

### c2 Comparisons

We next evaluated similarities and differences in over-represented biological pathways using the c2 gene set matrix, describing pathways. Compared to the c1 data set, fewer gene sets were similarly enriched in MM and MD comparisons. Three of four gene sets (proteasome, electron transport and oxidative phosphorylation pathways) were enriched for day 3 (compared to day 9) and zone II (compared to zone I) (Table S5–1,2, see supplemental data). Only two categories were similarly enriched between the MM and MD data sets in the 9 vs. 15, II vs. III comparisons. They belonged to the fatty acid and cytokine categories, both of which help modulate developmental processes by inhibiting chondrocyte hypertrophy and promoting prostaglandin synthesis [77]–[79]. The remaining 15 gene sets exhibited different enrichment patterns that, with the exception of the proteasome pathway (which was similarly enriched in zone III and day 9), showed similar enrichment in zone II and day 15 of MM culture (Table S5–3,4, see supplemental data). Thus, in both cases the enrichment patterns did not show the expected similarities between the MM AND MD data sets.

The day 3 vs. 15 and zone I vs. III fit best with our predictions for high similarity between the MM and MD data sets. All three gene sets showed correlation with day 15 MM and zone III of the tibia (Table S5–5,6, see supplemental data). The three gene sets represented in this comparison included the T-cell differentiation pathway, matrix metalloproteinases and reactive oxygen species.

These data indicate that there are some major gene expression differences between these experimental models. In addition, conservation between the MM day 3 vs. 9 and the MD zones I vs. II gene lists was poor. The day 9 vs. 15 and zone II vs. III pathways showed the highest similarity between day 15 of MM and zone II of MD, opposite to expectations. This result implicates enriched pathways in both hypertrophy and mineralization, which is not inconceivable, since these processes closely follow each other temporally. The observed enrichment pattern suggests that the ability of micromass cultures to recapitulate normal biological pathways is limited. The potential effects of enzymatic digestion, which disrupts and re-establishes the cell-cell and cell-matrix interactions in culture may account for this finding. Additionally, the inherent cellular heterogeneity persisting in the later stage chondrocyte cultures may be responsible for the apparent correlation between day 15 and zone II of MD. Specifically, chondrocytes in the centers of the micromass cultures and immature cells lining the periphery of the cartilage nodules even after 15 days of culture might additionally be responsible for this effect. Altogether these data suggest that the experimental system used may have a significant bearing on resolution of gene expression between different zones of the growth plate and also the biological pathways regulated in cartilage.

### c3 Comparisons

We next evaluated the similarities between the c3 data set that incorporates information about different documented regulatory motifs. The 3 vs. 9, I vs. II comparison identified 11 gene sets that were enriched on day 3 of MM and in zone I of MD, as expected (Table S6–1,2, see supplemental data). Five gene sets showed enrichment in day 3 of MM and zone II of MD. The remaining 4 gene sets were enriched in day 9 of MM and zone I of MD. All gene sets in the 9 vs. 15, II vs. III comparisons yielded similar gene sets between the two experimental methods. Eight gene sets were enriched in day 9 of MM and zone II of MD, while the other 12 were enriched at day 15 of MM and in zone III of the MD (Table S6–3,4, see supplemental data). In the final comparison between day 3 vs. 15 and zone I vs. III, 6 gene sets were enriched in day 3 of MM and zone I of the growth plate, 10 gene sets were enriched in day 15 of MM and zone III in the MD and 4 gene sets showed the opposing enrichment in day 3 MM and zone III of MD (Table S6–5,6, see supplemental data). Overall, regulatory motifs were well conserved between the MM and MD data sets, which could ideally provide clues into the identity of important chondrocyte regulatory molecules.

In conclusion, this study evaluates gene expression changes between three different zones of a growing tibia. Gene Ontology annotations show that similar functional categories are being modulated throughout chondrocyte maturation, namely genes related to development, ECM and transporter activity. GSEA enrichment of user-defined gene sets yielded several functional categories including ECM, growth factors, angiogenesis, chemokines and matrix metalloproteinases.

Interestingly, the “chaperone activity” category was enriched in the prehypertrophic/hypertrophic zone II when compared either with zone I or zone III. Recent studies have been investigated the role of endoplasmatic reticulum (ER) stress and unfolded protein response (UPR) in connective tissue diseases and particularly in chondrodysplasias. For example, ER stress was elevated in hypertrophic chondrocytes in a mouse model of metaphyseal chondrodysplasia type Schmid (MCDS) [80], folding of COMP was abnormal in pseudoachondroplasia and multiple epiphyseal dysplasia [81], [82] and *Bbf2h7* (an ER-resident transcription factor) KO mice showed severe chondrodysplasia with abnormal proliferative and hypertrophic zones [83]. These recent data show the importance of proper folding of secreted proteins – and by extension a role of chaperone proteins asisting with protein folding - in cartilage development. The enrichement of chaperone gene expression in the prehypertrophic/hypertrophic zone II of the growth plate makes this category a very exciting new pathway to investigate in cartilage development and particulalry in hypertrophic chondrocyte differentiation.

In addition, this study compared gene expression in micromass cultures, an in vitro model to study chondrocyte differentiation, with gene expression in growing endochondral bones in vivo. Parallel analysis in which gene expression in the MD array was compared to the MM array showed that while cytogenetic loci, some pathways and most motifs showed similar changes in the different models of chondrocyte development, individual markers exhibiting the largest gene expression changes in each data set were poorly conserved, with the exception of well known matrix markers and a few other genes. Pathway analysis also demonstrated limited similarities between MM and MD data sets, with the best fit for gene set correlated with day 15 and zone III and categories involved in angiogenesis and matrix remodeling.

While the micromass culture system is able to recapitulate expression of the major markers of chondrocyte gene expression, subtle, physiologically relevant differences could be overlooked in this model. Feasibility however, dictates that cell culture models are still an important means of evaluating cartilage gene expression. The challenge will be to design these models in a way in which the integrity of the tissue can be preserved, to quantify limitations in the models and, where possible, to make the transition to *in vivo* systems.

## Supporting Information

Table S1 GSEA analysis of comparisons between zones I and II of microdissected tibiae.
(0.33 MB DOC)

Table S2 GSEA analysis of comparisons between zones II and III of microdissected tibiae.
(0.25 MB DOC)

Table S3 GSEA analysis of comparisons between zones I and II of microdissected tibiae.
(0.45 MB DOC)

Table S4 GSEA enrichment of micromass culture data using c1 gene sets.
(0.15 MB DOC)

Table S5 GSEA enrichment of micromass culture data using c2 gene sets.
(0.13 MB DOC)

Table S6 GSEA enrichment of micromass culture data using c3 gene sets.
(0.27 MB DOC)
