# Supplementary material for: Genome-Wide Analyses of Gene Expression during Mouse Endochondral Ossification
Source: PLoS One. 2010 Jan 13;5(1):e8693. doi: 10.1371/journal.pone.0008693 (PMC2805713; doi:10.1371/journal.pone.0008693)
Supplement: Table S1 — GSEA analysis of comparisons between zones I and II of microdissected tibiae. (0.33 MB DOC) [file pone.0008693.s001.doc]

**Table S1-1. GSEA analysis of comparisons between zones I and II of microdissected tibiae.**

| NAME | SIZE | ES | NES | NOM p-val | FDR q-val |
| --- | --- | --- | --- | --- | --- |
| ECM | 228 | 0.524 | 1.874 | 0.000 | 0.006 |
| GROWTH FACTOR | 106 | 0.551 | 1.823 | 0.000 | 0.007 |
| 9 VS15 2 | 496 | 0.464 | 1.765 | 0.000 | 0.012 |
| 3VS9 1 | 495 | 0.458 | 1.738 | 0.000 | 0.013 |
| IGF | 48 | 0.586 | 1.724 | 0.003 | 0.013 |
| 3VS9 5 | 495 | 0.454 | 1.718 | 0.000 | 0.012 |
| GROWTH FACTOR RECEPTOR | 327 | 0.462 | 1.713 | 0.000 | 0.011 |
| 9VS15 1 | 497 | 0.443 | 1.682 | 0.000 | 0.014 |
| ANGIOGEN | 57 | 0.555 | 1.679 | 0.003 | 0.013 |
| HEPARIN BINDING | 37 | 0.583 | 1.633 | 0.014 | 0.021 |
| BRAIN | 379 | 0.435 | 1.619 | 0.000 | 0.022 |
| GTPASE ACTIVITY | 73 | 0.516 | 1.614 | 0.006 | 0.022 |
| 9VS15 3 | 497 | 0.421 | 1.595 | 0.000 | 0.025 |
| 3VS9 3 | 495 | 0.414 | 1.570 | 0.000 | 0.029 |
| 3VS15 1 | 497 | 0.410 | 1.553 | 0.000 | 0.032 |
| CHROMATIN/HDAC | 37 | 0.551 | 1.543 | 0.029 | 0.033 |
| FGF | 64 | 0.503 | 1.537 | 0.016 | 0.033 |
| CHEMOKINE | 31 | 0.566 | 1.516 | 0.036 | 0.038 |
| 3VS15 2 | 497 | 0.400 | 1.515 | 0.000 | 0.037 |
| 2 DNABIND | 449 | 0.399 | 1.499 | 0.000 | 0.041 |
| CHAPERONE | 81 | -0.476 | -1.721 | 0.002 | 0.074 |
| PHOSPHATASE | 473 | -0.310 | -1.404 | 0.000 | 0.382 |
| METABOLISM | 196 | -0.331 | -1.367 | 0.006 | 0.324 |
| TGFB | 45 | -0.414 | -1.349 | 0.085 | 0.273 |
| CATALYTIC | 245 | -0.307 | -1.312 | 0.024 | 0.274 |
| OBL OCLAST | 16 | -0.497 | -1.290 | 0.168 | 0.259 |
| BONE | 116 | -0.333 | -1.287 | 0.065 | 0.227 |
| CYTOKINE | 127 | -0.324 | -1.259 | 0.070 | 0.234 |
| NEG APOPTOSIS | 50 | -0.367 | -1.218 | 0.172 | 0.264 |
| LIVER 2 | 260 | -0.270 | -1.148 | 0.114 | 0.353 |
| WNT | 53 | -0.320 | -1.071 | 0.339 | 0.495 |
| HZ HORTON | 407 | -0.234 | -1.044 | 0.306 | 0.531 |
| PZ HORTON | 413 | -0.231 | -1.030 | 0.329 | 0.533 |
| TUMOR SUPPRESSOR | 48 | -0.312 | -1.010 | 0.438 | 0.549 |
| LIVER 1 | 260 | -0.221 | -0.944 | 0.640 | 0.705 |
| TNF RECEPTOR | 69 | -0.260 | -0.930 | 0.599 | 0.700 |
| ELECTRON TRANSPORT | 40 | -0.285 | -0.902 | 0.616 | 0.727 |
| FKBP | 33 | -0.284 | -0.865 | 0.660 | 0.761 |

* negative values indicate correlation of enrichment scores (ES) with zone II

NES=normalized enrichment score, FDR=false discovery rate

**Table S1-2. ECM transcripts enriched in zone I vs. II comparisons.**

| HUGO gene symbol | RANK | RMS | RES |
| --- | --- | --- | --- |
| Kera | 5 | 0.950 | 0.023 |
| Cspg2 | 16 | 0.844 | 0.044 |
| Ltbp1 | 36 | 0.734 | 0.061 |
| Fbn2 | 37 | 0.732 | 0.079 |
| Bc063774 | 38 | 0.726 | 0.097 |
| Lamb1-1 | 44 | 0.715 | 0.115 |
| Col14a1 | 48 | 0.708 | 0.132 |
| Dpt | 55 | 0.698 | 0.149 |
| Aspn | 68 | 0.665 | 0.165 |
| Ntng1 | 71 | 0.659 | 0.181 |
| Ptn | 98 | 0.604 | 0.195 |
| Postn | 105 | 0.594 | 0.210 |
| Adamts15 | 126 | 0.567 | 0.223 |
| Col4a2 | 130 | 0.563 | 0.236 |
| Fbn1 | 147 | 0.543 | 0.249 |
| Col8a2 | 149 | 0.540 | 0.263 |
| Mfap4 | 196 | 0.483 | 0.272 |
| Bgn | 200 | 0.476 | 0.284 |
| Emilin2 | 210 | 0.469 | 0.295 |
| Gpc2 | 232 | 0.449 | 0.305 |
| Eln | 249 | 0.434 | 0.315 |
| Col4a1 | 256 | 0.428 | 0.325 |
| Fbln1 | 263 | 0.424 | 0.336 |
| Tek | 295 | 0.402 | 0.344 |
| Ltbp3 | 304 | 0.398 | 0.354 |
| Col8a1 | 315 | 0.392 | 0.363 |
| Col5a1 | 348 | 0.375 | 0.371 |
| Mmp11 | 372 | 0.368 | 0.379 |
| Fmod | 383 | 0.363 | 0.387 |
| Itga9 | 392 | 0.359 | 0.396 |
| Emid2 | 414 | 0.351 | 0.403 |
| Col18a1 | 515 | 0.315 | 0.406 |
| Dcn | 532 | 0.310 | 0.413 |
| Adamts5 | 550 | 0.307 | 0.420 |
| Mmp14 | 559 | 0.304 | 0.427 |
| Col3a1 | 596 | 0.296 | 0.432 |
| Col6a2 | 615 | 0.291 | 0.439 |
| Adamtsl1 | 620 | 0.290 | 0.446 |
| Col5a2 | 628 | 0.288 | 0.452 |
| Cd44 | 629 | 0.288 | 0.460 |
| Mfap5 | 721 | 0.266 | 0.462 |

**Table S1-3. ECM transcripts enriched in zone I vs. II comparisons.**

| HUGO gene symbol | RANK | RMS | RES |
| --- | --- | --- | --- |
| Mfap2 | 785 | 0.253 | 0.465 |
| Emilin1 | 797 | 0.250 | 0.470 |
| Timp2 | 870 | 0.236 | 0.473 |
| Matn4 | 871 | 0.235 | 0.478 |
| Smoc1 | 908 | 0.228 | 0.482 |
| Col6a1 | 915 | 0.228 | 0.488 |
| Col17a1 | 931 | 0.225 | 0.493 |
| Lamb3 | 948 | 0.223 | 0.497 |
| Adamts1 | 1127 | 0.200 | 0.493 |
| Mmp2 | 1169 | 0.195 | 0.496 |
| Tnfrsf11b | 1173 | 0.195 | 0.501 |
| Gpc6 | 1217 | 0.190 | 0.503 |
| Adam12 | 1222 | 0.189 | 0.508 |
| Ltbp4 | 1241 | 0.187 | 0.511 |
| Col16a1 | 1290 | 0.182 | 0.514 |
| Timp3 | 1340 | 0.177 | 0.515 |
| Ntn1 | 1469 | 0.166 | 0.513 |
| Adamts2 | 1502 | 0.164 | 0.516 |
| Fbln2 | 1561 | 0.160 | 0.517 |
| B4galt1 | 1584 | 0.159 | 0.520 |
| Lamc1 | 1701 | 0.149 | 0.517 |
| Chad | 1718 | 0.148 | 0.520 |
| Adamts10 | 1719 | 0.148 | 0.524 |

RANK= position of genes in the context of the ranked list of array genes

RMS = the ranked metric score

RES = the running enrichment score

note: positive RES indicates enrichment in zone I

**Table S1-4. Growth factor transcripts enriched in zone I vs. II comparisons.**

| HUGO gene symbol | RANK | RMS | RES |
| --- | --- | --- | --- |
| Nov | 8 | 0.920 | 0.071 |
| Cxcl12 | 32 | 0.760 | 0.130 |
| Ptn | 98 | 0.604 | 0.173 |
| Vegfc | 129 | 0.564 | 0.216 |
| Kitlg | 163 | 0.519 | 0.255 |
| Ntf3 | 208 | 0.471 | 0.289 |
| Fgf18 | 224 | 0.458 | 0.324 |
| Fgf7 | 258 | 0.427 | 0.356 |
| Gdf5 | 283 | 0.410 | 0.387 |
| Mdk | 338 | 0.379 | 0.414 |
| Igf1 | 364 | 0.370 | 0.441 |
| Bmp5 | 701 | 0.271 | 0.445 |
| Vegfb | 761 | 0.259 | 0.463 |
| Btc | 790 | 0.252 | 0.481 |
| Inhba | 828 | 0.243 | 0.498 |
| Fgf13 | 897 | 0.231 | 0.513 |
| Hbegf | 983 | 0.217 | 0.525 |
| Tgfa | 1365 | 0.174 | 0.520 |
| Gmfg | 1410 | 0.171 | 0.531 |
| Tgfb3 | 1454 | 0.168 | 0.542 |
| Pgf | 1517 | 0.163 | 0.551 |

RANK= position of genes in the context of the ranked list of array genes

RMS = the ranked metric score

RES = the running enrichment score

note: positive RES indicates enrichment in zone I

**Table S1-5. Transcripts involved in angiogenesis enriched in zone I vs. II comparisons.**

| HUGO gene symbol | RANK | RMS | RES |
| --- | --- | --- | --- |
| Tbx4 | 0 | 1.106 | 0.144 |
| Vegfc | 129 | 0.564 | 0.210 |
| Bai3 | 279 | 0.412 | 0.256 |
| Tek | 295 | 0.402 | 0.308 |
| Bai2 | 393 | 0.359 | 0.350 |
| Col18a1 | 515 | 0.315 | 0.384 |
| Arts-1 | 678 | 0.276 | 0.412 |
| Vegfb | 761 | 0.259 | 0.442 |
| Kdr | 850 | 0.240 | 0.468 |
| Angpt1 | 990 | 0.216 | 0.490 |
| Tie1 | 1066 | 0.207 | 0.513 |
| Pgf | 1517 | 0.163 | 0.511 |
| Smad5 | 1637 | 0.154 | 0.526 |
| Wasf2 | 2068 | 0.128 | 0.521 |
| Figf | 2155 | 0.124 | 0.532 |
| Angpt2 | 2182 | 0.123 | 0.547 |
| Mapk7 | 2628 | 0.103 | 0.538 |
| Eng | 2769 | 0.098 | 0.544 |
| Egfl7 | 2806 | 0.096 | 0.555 |

RANK= position of genes in the context of the ranked list of array genes

RMS = the ranked metric score

RES = the running enrichment score

note: positive RES indicates enrichment in zone I

**Table S1-6. Chemokine transcripts enriched in zone I vs. II comparisons.**

| HUGO gene symbol | RANK | RMS | RES |
| --- | --- | --- | --- |
| Cxcl12 | 32 | 0.76039553 | 0.25126913 |
| Pf4 | 518 | 0.3141461 | 0.33150357 |
| Ppbp | 834 | 0.24169841 | 0.39614013 |
| Cxcl14 | 1039 | 0.21037415 | 0.45590627 |
| Ccl12 | 1444 | 0.16845182 | 0.49173772 |
| Ccl27 | 1529 | 0.16211791 | 0.54145247 |
| Cxcl1 | 1940 | 0.13469739 | 0.5657591 |

RANK= position of genes in the context of the ranked list of array genes

RMS = the ranked metric score

RES = the running enrichment score

note: positive RES indicates enrichment in zone I

**Table S1-7. Transcripts involved in chaperone activity enriched in zone I vs. II comparisons.**

| HUGO gene symbol | RANK | RMS | RES |
| --- | --- | --- | --- |
| Dnajc7 | 13687 | -0.041 | -0.468 |
| Thoc4 | 13777 | -0.042 | -0.466 |
| Ndufaf1 | 13866 | -0.043 | -0.463 |
| Cct2 | 13891 | -0.043 | -0.456 |
| Tbca | 13974 | -0.043 | -0.453 |
| Hspe1 | 14136 | -0.045 | -0.453 |
| Stch | 14183 | -0.045 | -0.448 |
| Ckap1 | 14190 | -0.045 | -0.440 |
| Clgn | 14253 | -0.046 | -0.435 |
| 4833428e21rik | 14320 | -0.046 | -0.431 |
| Ywhaq | 14408 | -0.047 | -0.427 |
| Dnajb9 | 14558 | -0.049 | -0.426 |
| 1700010a06rik | 14709 | -0.050 | -0.425 |
| Hspa1b | 14895 | -0.051 | -0.425 |
| Hspa9a | 14912 | -0.052 | -0.417 |
| Trap1 | 14930 | -0.052 | -0.409 |
| St13 | 14998 | -0.052 | -0.403 |
| Dnajc4 | 15028 | -0.053 | -0.395 |
| Pfdn5 | 15066 | -0.053 | -0.388 |
| Cct7 | 15470 | -0.057 | -0.398 |
| Cdc37 | 15489 | -0.057 | -0.389 |
| Dnaja3 | 15503 | -0.057 | -0.380 |
| Vbp1 | 15641 | -0.059 | -0.377 |
| D230036h06rik | 15909 | -0.062 | -0.379 |
| Calr | 16016 | -0.063 | -0.374 |
| Cct8 | 16328 | -0.067 | -0.378 |
| Ccs | 16504 | -0.069 | -0.375 |
| Hyou1 | 16583 | -0.070 | -0.366 |
| Mkks | 17444 | -0.083 | -0.395 |
| Atox1 | 18093 | -0.099 | -0.410 |
| Pfdn1 | 18244 | -0.102 | -0.400 |
| Dnajc1 | 18321 | -0.105 | -0.386 |
| Grpel1 | 18572 | -0.116 | -0.378 |
| Dnajb11 | 19009 | -0.140 | -0.376 |
| Dnaja4 | 19248 | -0.163 | -0.359 |
| Dnajb12 | 19408 | -0.189 | -0.335 |
| Uxt | 19690 | -0.260 | -0.304 |
| Dnajb10 | 19779 | -0.305 | -0.255 |
| H2-Dmb1 | 19781 | -0.305 | -0.203 |
| H2-Dma | 19863 | -0.355 | -0.145 |
| Gcnt2 | 20027 | -0.889 | 0.001 |

RANK= position of genes in the context of the ranked list of array

genes; RMS = the ranked metric score; RES = the running

enrichment score; note: negative RES indicates enrichment in zone II

**Table S1-8. Bone transcripts enriched in zone I vs. II comparisons.**

| HUGO gene symbol | RANK | RMS | RES |
| --- | --- | --- | --- |
| Cpeb4 | 18680 | -0.121 | -0.322 |
| Bb166591 | 18696 | -0.122 | -0.314 |
| Fn1 | 18802 | -0.128 | -0.311 |
| Bzrp | 19217 | -0.159 | -0.321 |
| Atxn7l4 | 19274 | -0.167 | -0.312 |
| Bmp4 | 19696 | -0.261 | -0.316 |
| Bmp7 | 19780 | -0.305 | -0.299 |
| Akp2 | 19896 | -0.379 | -0.279 |
| Bmp8a | 19993 | -0.651 | -0.239 |
| Pthr1 | 20001 | -0.669 | -0.194 |
| Tnfsf11 | 20004 | -0.672 | -0.149 |
| Mbp | 20025 | -0.883 | -0.089 |
| Ibsp | 20040 | -1.326 | 0.000 |

RANK= position of genes in the context of the ranked list of array genes

RMS = the ranked metric score

RES = the running enrichment score

note: negative RES indicates enrichment in zone II

**Table S1-9 Transcripts involved in chaperone activity enriched in zone I vs. II comparisons.**

| HUGO gene symbol | RANK | RMS | RES |
| --- | --- | --- | --- |
| Dnajc7 | 13687 | -0.041 | -0.468 |
| Thoc4 | 13777 | -0.042 | -0.466 |
| Ndufaf1 | 13866 | -0.043 | -0.463 |
| Cct2 | 13891 | -0.043 | -0.456 |
| Tbca | 13974 | -0.043 | -0.453 |
| Hspe1 | 14136 | -0.045 | -0.453 |
| Stch | 14183 | -0.045 | -0.448 |
| Ckap1 | 14190 | -0.045 | -0.440 |
| Clgn | 14253 | -0.046 | -0.435 |
| 4833428e21rik | 14320 | -0.046 | -0.431 |
| Ywhaq | 14408 | -0.047 | -0.427 |
| Dnajb9 | 14558 | -0.049 | -0.426 |
| 1700010a06rik | 14709 | -0.050 | -0.425 |
| Hspa1b | 14895 | -0.051 | -0.425 |
| Hspa9a | 14912 | -0.052 | -0.417 |
| Trap1 | 14930 | -0.052 | -0.409 |
| St13 | 14998 | -0.052 | -0.403 |
| Dnajc4 | 15028 | -0.053 | -0.395 |
| Pfdn5 | 15066 | -0.053 | -0.388 |
| Cct7 | 15470 | -0.057 | -0.398 |
| Cdc37 | 15489 | -0.057 | -0.389 |
| Dnaja3 | 15503 | -0.057 | -0.380 |
| Vbp1 | 15641 | -0.059 | -0.377 |
| D230036h06rik | 15909 | -0.062 | -0.379 |
| Calr | 16016 | -0.063 | -0.374 |
| Cct8 | 16328 | -0.067 | -0.378 |
| Ccs | 16504 | -0.069 | -0.375 |
| Hyou1 | 16583 | -0.070 | -0.366 |
| Mkks | 17444 | -0.083 | -0.395 |
| Atox1 | 18093 | -0.099 | -0.410 |
| Pfdn1 | 18244 | -0.102 | -0.400 |
| Dnajc1 | 18321 | -0.105 | -0.386 |
| Grpel1 | 18572 | -0.116 | -0.378 |
| Dnajb11 | 19009 | -0.140 | -0.376 |
| Dnaja4 | 19248 | -0.163 | -0.359 |
| Dnajb12 | 19408 | -0.189 | -0.335 |
| Uxt | 19690 | -0.260 | -0.304 |
| Dnajb10 | 19779 | -0.305 | -0.255 |
| H2-Dmb1 | 19781 | -0.305 | -0.203 |
| H2-Dma | 19863 | -0.355 | -0.145 |
| Gcnt2 | 20027 | -0.889 | 0.001 |

RANK= position of genes in the context of the ranked list of array

genes; RMS = the ranked metric score; RES = the running

enrichment score; note: negative RES indicates enrichment in zone II
