# Supplementary material for: Genome-Wide Analyses of Gene Expression during Mouse Endochondral Ossification
Source: PLoS One. 2010 Jan 13;5(1):e8693. doi: 10.1371/journal.pone.0008693 (PMC2805713; doi:10.1371/journal.pone.0008693)
Supplement: Table S2 — GSEA analysis of comparisons between zones II and III of microdissected tibiae. (0.25 MB DOC) [file pone.0008693.s002.doc]

**Table S2-1. GSEA analysis of comparisons between zones II and III of microdissected tibiae.**

| NAME | SIZE | ES | NES | NOM p-val | FDR q-val |
| --- | --- | --- | --- | --- | --- |
| CARTILAGE | 28 | 0.579 | 1.742 | 0.007 | 0.032 |
| CHAPERONE | 81 | 0.391 | 1.467 | 0.017 | 0.166 |
| METABOLISM | 196 | 0.335 | 1.411 | 0.003 | 0.163 |
| GLUCONEOGEN | 31 | 0.452 | 1.385 | 0.075 | 0.145 |
| CATALYTIC | 245 | 0.274 | 1.191 | 0.054 | 0.372 |
| TUMOR SUPPRESSOR | 48 | 0.297 | 1.006 | 0.436 | 0.882 |
| WNT | 53 | 0.274 | 0.939 | 0.566 | 1.000 |
| HZHORTON.FARNUM | 407 | 0.201 | 0.936 | 0.766 | 0.911 |
| HORMONE | 75 | 0.252 | 0.933 | 0.607 | 0.820 |
| FKBP | 33 | 0.284 | 0.894 | 0.632 | 0.840 |
| PZHORTON.FARNUM | 413 | 0.196 | 0.892 | 0.924 | 0.766 |
| HEPATOCYTE | 19 | 0.307 | 0.839 | 0.690 | 0.804 |
| HEPARIN BIND | 37 | -0.730 | -2.010 | 0.000 | 0.000 |
| ANGIOGEN | 57 | -0.645 | -1.897 | 0.000 | 0.001 |
| CHEMOKINE | 31 | -0.686 | -1.816 | 0.001 | 0.005 |
| 9 VS15 2 | 496 | -0.498 | -1.807 | 0.000 | 0.005 |
| 9VS15 1 | 497 | -0.492 | -1.792 | 0.000 | 0.004 |
| 9VS15 3 | 497 | -0.491 | -1.784 | 0.000 | 0.004 |
| 3VS9 1 | 495 | -0.481 | -1.748 | 0.000 | 0.005 |
| 3VS9 5 | 495 | -0.479 | -1.738 | 0.000 | 0.006 |
| 3VS9 3 | 495 | -0.478 | -1.737 | 0.000 | 0.005 |
| 3VS15 5 | 496 | -0.473 | -1.720 | 0.000 | 0.006 |
| ECM | 228 | -0.493 | -1.717 | 0.000 | 0.006 |
| ADIPOSE | 70 | -0.558 | -1.716 | 0.002 | 0.005 |
| BLOOD | 111 | -0.518 | -1.689 | 0.000 | 0.007 |
| GF RECEPTOR | 327 | -0.472 | -1.674 | 0.000 | 0.008 |
| 3VS15 1 | 497 | -0.455 | -1.653 | 0.000 | 0.010 |
| CART 3 | 352 | -0.458 | -1.639 | 0.000 | 0.011 |
| 3VS15 3 | 496 | -0.449 | -1.633 | 0.000 | 0.011 |
| KINASE 3 | 227 | -0.469 | -1.631 | 0.000 | 0.011 |
| TF REPRESS | 55 | -0.552 | -1.629 | 0.004 | 0.010 |
| 3VS9 2 | 494 | -0.449 | -1.626 | 0.000 | 0.010 |

* negative values indicate correlation of scores with zone III

ES=enrichment score

NES=normalized enrichment score

FDR=false discovery rate

**Table S2-2 Transcripts involved in chaperone activity enriched in II vs. III comparisons.**

| HUGO gene symbol | RANK | RMS | RES |
| --- | --- | --- | --- |
| H2-Dma | 26 | 0.834 | 0.112 |
| H2-Dmb1 | 110 | 0.533 | 0.181 |
| Gcnt2 | 127 | 0.504 | 0.249 |
| Dnajb10 | 429 | 0.299 | 0.274 |
| Uxt | 717 | 0.229 | 0.291 |
| Ppih | 1081 | 0.186 | 0.298 |
| Dnajc12 | 1218 | 0.172 | 0.315 |
| H2-Dmb2 | 1255 | 0.169 | 0.336 |
| Thoc4 | 1543 | 0.148 | 0.342 |
| Hspa1b | 1621 | 0.143 | 0.358 |
| Bag1 | 1697 | 0.140 | 0.373 |
| Stch | 1824 | 0.134 | 0.385 |
| Calr3 | 2048 | 0.125 | 0.391 |

RMS = the ranked metric score

RES = the running enrichment score

note: positive RES indicates enrichment in zone II

**Table S2-3. Metabolic transcripts enriched in zone II vs. III comparisons.**

| HUGO gene symbol | RANK | RMS | RES |
| --- | --- | --- | --- |
| Gcat | 112 | 0.532 | 0.019 |
| Sc5dl | 115 | 0.523 | 0.043 |
| Cbr2 | 137 | 0.486 | 0.064 |
| Rdh11 | 182 | 0.439 | 0.082 |
| Uap1 | 235 | 0.388 | 0.098 |
| Plod2 | 267 | 0.366 | 0.113 |
| Pyp | 286 | 0.361 | 0.129 |
| Rdh12 | 289 | 0.359 | 0.145 |
| Ugp2 | 336 | 0.335 | 0.158 |
| P4ha2 | 344 | 0.331 | 0.173 |
| Sptlc2 | 351 | 0.328 | 0.188 |
| Adhfe1 | 384 | 0.315 | 0.201 |
| Hibch | 390 | 0.312 | 0.215 |
| Acas2 | 424 | 0.3 | 0.227 |
| Fthfd | 513 | 0.276 | 0.236 |
| Fzd1 | 690 | 0.234 | 0.238 |
| Plod1 | 696 | 0.233 | 0.248 |
| Pecr | 740 | 0.225 | 0.256 |
| Bpgm | 823 | 0.214 | 0.262 |
| Hsd17b7 | 849 | 0.209 | 0.27 |
| Amacr | 873 | 0.206 | 0.279 |
| Mthfs | 888 | 0.205 | 0.288 |
| Dhrs7 | 1019 | 0.192 | 0.29 |
| Mvk | 1125 | 0.181 | 0.293 |
| Aacs | 1239 | 0.17 | 0.295 |
| 2310057D15RIK | 1322 | 0.163 | 0.298 |
| Gstm5 | 1413 | 0.156 | 0.301 |
| Tpi1 | 1429 | 0.155 | 0.308 |
| Plod3 | 1436 | 0.155 | 0.314 |
| 1810010G06RIK | 1602 | 0.144 | 0.313 |
| 2410012M04RIK | 1689 | 0.141 | 0.315 |
| 0610042E07RIK | 1782 | 0.136 | 0.316 |
| 933406E20RIK | 1819 | 0.134 | 0.321 |
| Hsd17b1 | 1909 | 0.13 | 0.322 |
| Pmm1 | 1960 | 0.129 | 0.326 |
| Gstm2 | 2313 | 0.117 | 0.313 |

**Table S2-4. Metabolic transcripts enriched in zone II vs. III comparisons.**

| HUGO gene symbol | RANK | RMS | RES |
| --- | --- | --- | --- |
| Decr1 | 2351 | 0.115 | 0.317 |
| Dhrs2 | 2434 | 0.113 | 0.318 |
| 2010321J07RIK | 2523 | 0.11 | 0.319 |
| Mut | 2549 | 0.11 | 0.322 |
| Mccc1 | 2550 | 0.11 | 0.328 |
| Dhrs2 | 2434 | 0.113 | 0.318 |
| 2010321J07RIK | 2523 | 0.11 | 0.319 |
| Mut | 2549 | 0.11 | 0.322 |
| Mccc1 | 2550 | 0.11 | 0.328 |
| Slc38a4 | 2910 | 0.101 | 0.314 |
| 2610209N15RIK | 2933 | 0.101 | 0.318 |
| Dhdds | 2949 | 0.101 | 0.322 |
| Bcat2 | 3013 | 0.099 | 0.323 |
| Apba2bp | 3027 | 0.099 | 0.327 |
| PFKFB1 | 3076 | 0.098 | 0.329 |
| HSD17B2 | 3174 | 0.096 | 0.329 |
| DCT | 3211 | 0.096 | 0.331 |
| TECTA | 3231 | 0.095 | 0.335 |

RANK= position of genes in the context of the ranked list of array genes

RMS = the ranked metric score

RES = the running enrichment score

note: positive RES indicates enrichment in zone II

**Table S2-5. Transcripts involved in gluconeogenesis enriched in zone II vs. III comparisons.**

| HUGO gene symbol | RANK | RMS | RES |
| --- | --- | --- | --- |
| Aldoc | 151 | 0.476 | 0.125 |
| Pfkp | 785 | 0.218 | 0.154 |
| Eno2 | 1034 | 0.190 | 0.195 |
| Ldhc | 1211 | 0.173 | 0.234 |
| Tpi1 | 1429 | 0.155 | 0.266 |
| Pgk2 | 1535 | 0.149 | 0.303 |
| Hk2 | 1587 | 0.145 | 0.341 |
| Pfkm | 1941 | 0.129 | 0.359 |
| Pfkl | 2594 | 0.109 | 0.357 |
| Fbp1 | 2704 | 0.106 | 0.380 |
| Pklr | 2707 | 0.105 | 0.410 |
| Eno1 | 2975 | 0.100 | 0.424 |
| Gck | 2983 | 0.100 | 0.452 |

RANK= position of genes in the context of the ranked list of array genes

RMS = the ranked metric score

RES = the running enrichment score

note: positive RES indicates enrichment in zone II

**Table S2-6. Transcripts involved in heparin binding enriched in zone II vs. III comparisons.**

| HUGO gene symbol | RANK | RMS | RES |
| --- | --- | --- | --- |
| Vegf | 18426 | -0.213 | -0.700 |
| Fn1 | 18476 | -0.218 | -0.672 |
| Hbegf | 18539 | -0.224 | -0.643 |
| Lpl | 18592 | -0.232 | -0.613 |
| Adamts1 | 18761 | -0.253 | -0.586 |
| Pf4 | 18798 | -0.258 | -0.552 |
| Thbs2 | 18849 | -0.265 | -0.517 |
| Ncam1 | 19032 | -0.299 | -0.484 |
| Mdk | 19634 | -0.494 | -0.445 |
| Serpine2 | 19635 | -0.494 | -0.376 |
| Gpnmb | 19756 | -0.567 | -0.302 |
| Lipc | 19822 | -0.625 | -0.218 |
| Apoe | 19947 | -0.804 | -0.111 |
| Serpind1 | 19960 | -0.828 | 0.004 |

RANK= position of genes in the context of the ranked list of array genes

RMS = the ranked metric score

RES = the running enrichment score

note: negative RES indicates enrichment in zone III

**Table S2-7. Angiogenic transcripts enriched in II vs. III comparisons.**

| HUGO gene symbol | RANK | RMS | RES |
| --- | --- | --- | --- |
| Tnfaip2 | 17062 | -0.117 | -0.636 |
| Vegfb | 17204 | -0.123 | -0.633 |
| Mapk14 | 17305 | -0.128 | -0.628 |
| Ubp1 | 17320 | -0.129 | -0.619 |
| Serpine1 | 17354 | -0.131 | -0.610 |
| Smad5 | 17359 | -0.131 | -0.600 |
| Gna13 | 18211 | -0.194 | -0.628 |
| Epas1 | 18223 | -0.195 | -0.613 |
| Fzd5 | 18288 | -0.200 | -0.600 |
| Vegf | 18426 | -0.213 | -0.591 |
| 2010009l17rik | 18442 | -0.214 | -0.575 |
| Wasf2 | 18486 | -0.219 | -0.560 |
| Flt1 | 18570 | -0.228 | -0.546 |
| Nrp1 | 18692 | -0.244 | -0.533 |
| Eng | 18951 | -0.283 | -0.524 |
| Tbx4 | 19390 | -0.391 | -0.515 |
| Itgav | 19395 | -0.392 | -0.485 |
| Egfl7 | 19417 | -0.398 | -0.454 |
| Angpt1 | 19538 | -0.451 | -0.425 |
| Tbx1 | 19544 | -0.453 | -0.390 |
| Arts-1 | 19554 | -0.456 | -0.355 |
| Angpt2 | 19562 | -0.459 | -0.319 |
| Kdr | 19788 | -0.596 | -0.284 |
| Col18a1 | 19837 | -0.643 | -0.236 |
| Tie1 | 19852 | -0.658 | -0.185 |
| Tek | 19933 | -0.766 | -0.129 |
| Pgf | 19935 | -0.771 | -0.069 |
| Vegfc | 19997 | -0.951 | 0.002 |

RANK= position of genes in the context of the ranked list of array genes

RMS = the ranked metric score

RES = the running enrichment score

note: negative RES indicates enrichment in zone III

**Table S2-8. Chemokine transcripts enriched in zone II vs. III comparisons.**

| HUGO gene symbol | RANK | RMS | RES |
| --- | --- | --- | --- |
| Pf4 | 18798 | -0.258 | -0.628 |
| Ppbp | 19235 | -0.344 | -0.572 |
| Ccl6 | 19451 | -0.408 | -0.490 |
| Ccl9 | 19479 | -0.422 | -0.397 |
| Cxcl2 | 19691 | -0.521 | -0.290 |
| Ccl3 | 19710 | -0.535 | -0.170 |
| Cxcl12 | 19959 | -0.826 | 0.004 |

RANK= position of genes in the context of the ranked list of array genes

RMS = the ranked metric score

RES = the running enrichment score

note: negative RES indicates enrichment in zone III

**Table S2-9. Cartilage transcripts enriched in zone II vs. III comparisons.**

| HUGO gene symbol | RANK | RMS | RES |
| --- | --- | --- | --- |
| Sox9 | 66 | 0.641 | 0.105 |
| Lect1 | 80 | 0.593 | 0.205 |
| Mia1 | 173 | 0.449 | 0.277 |
| Hapln1 | 256 | 0.372 | 0.336 |
| Matn1 | 330 | 0.338 | 0.389 |
| Comp | 457 | 0.291 | 0.432 |
| Cart1 | 522 | 0.274 | 0.476 |
| Agc1 | 528 | 0.273 | 0.522 |
| Bmpr1b | 816 | 0.215 | 0.544 |
| Hapln3 | 831 | 0.212 | 0.579 |

RANK= position of genes in the context of the ranked list of array genes

RMS = the ranked metric score

RES = the running enrichment score

note: positive RES indicates enrichment in zone II
