# Supplementary material for: Genome-Wide Analyses of Gene Expression during Mouse Endochondral Ossification
Source: PLoS One. 2010 Jan 13;5(1):e8693. doi: 10.1371/journal.pone.0008693 (PMC2805713; doi:10.1371/journal.pone.0008693)
Supplement: Table S3 — GSEA analysis of comparisons between zones I and II of microdissected tibiae. (0.45 MB DOC) [file pone.0008693.s003.doc]

**Table S3-1. GSEA analysis of comparisons between zones I and II of microdissected tibiae**.

| NAME | SIZE | ES | NES | NOM p-val | FDR q-val |
| --- | --- | --- | --- | --- | --- |
|  |  |  |  |  |  |
| CARTILAGE | 28 | 0.551 | 1.449 | 0.059 | 0.381 |
| IGF | 48 | 0.460 | 1.371 | 0.064 | 0.351 |
| BRAIN REL | 379 | 0.321 | 1.251 | 0.020 | 0.539 |
| GLUCONEOGEN | 31 | 0.457 | 1.232 | 0.180 | 0.458 |
| MEMBRANE | 260 | 0.318 | 1.194 | 0.064 | 0.474 |
| MUSCLE | 198 | 0.304 | 1.112 | 0.188 | 0.682 |
| FGF RELATED | 64 | 0.356 | 1.111 | 0.259 | 0.589 |
| GROWTH FACTOR | 106 | 0.316 | 1.060 | 0.324 | 0.724 |
| BMPRELATED | 62 | 0.340 | 1.056 | 0.332 | 0.659 |
| STRUCTURE | 151 | 0.295 | 1.036 | 0.372 | 0.676 |
| METABOLISM | 196 | 0.271 | 0.993 | 0.466 | 0.804 |
| GTPASE ACTIV | 73 | 0.304 | 0.969 | 0.520 | 0.851 |
| HEPATOCYTE | 19 | 0.394 | 0.962 | 0.519 | 0.816 |
| NUCLEUS 2 | 494 | 0.241 | 0.958 | 0.655 | 0.773 |
| HORMONE | 75 | 0.298 | 0.951 | 0.569 | 0.747 |
| WNT | 53 | 0.312 | 0.931 | 0.573 | 0.773 |
| ACTIN CYTOSKEL | 38 | 0.305 | 0.850 | 0.717 | 0.931 |
| TUMOR SUPPRESSOR | 48 | 0.272 | 0.800 | 0.809 | 0.948 |
| CHAPERONE | 81 | 0.225 | 0.727 | 0.961 | 0.953 |
| BLOOD | 111 | -0.524 | -1.650 | 0.001 | 0.166 |
| 3VS9 6 | 276 | -0.472 | -1.649 | 0.000 | 0.083 |
| PHOSPHATASE | 473 | -0.450 | -1.648 | 0.000 | 0.057 |
| OBL OCLAST | 16 | -0.719 | -1.609 | 0.023 | 0.062 |
| CART 3 | 352 | -0.447 | -1.599 | 0.000 | 0.056 |
| 3VS15 3 | 496 | -0.434 | -1.591 | 0.000 | 0.051 |
| SUGAR BIND | 104 | -0.508 | -1.588 | 0.004 | 0.046 |
| 3VS15 5 | 496 | -0.433 | -1.585 | 0.000 | 0.041 |
| 9VS15 1 | 497 | -0.427 | -1.561 | 0.000 | 0.047 |
| BONE | 116 | -0.490 | -1.547 | 0.005 | 0.049 |
| TGFB | 45 | -0.563 | -1.546 | 0.021 | 0.045 |
| KINASE 3 | 227 | -0.448 | -1.539 | 0.002 | 0.044 |
| MAPKRELATED | 267 | -0.442 | -1.531 | 0.000 | 0.043 |
| ANGIOGEN | 57 | -0.530 | -1.525 | 0.021 | 0.043 |
| 9VS15 3 | 497 | -0.417 | -1.521 | 0.000 | 0.041 |
| HEPARIN BIND | 37 | -0.565 | -1.503 | 0.036 | 0.046 |
| RELATED APOPTOSIS | 311 | -0.425 | -1.502 | 0.001 | 0.043 |
| ERK RELATED | 40 | -0.548 | -1.495 | 0.030 | 0.044 |
| 3VS15 8 | 462 | -0.404 | -1.478 | 0.000 | 0.049 |
| LIVER 1 | 260 | -0.418 | -1.451 | 0.001 | 0.059 |

* negative values indicate correlation of scores with the II; ES=enrichment score ; NES=normalized enrichment score; FDR=false discovery rate

**Table S3-2. Transcripts expressed in blood enriched in zone I vs. III comparisons.**

| HUGO gene symbol | RANK | RMS | RES |
| --- | --- | --- | --- |
| Abcb1a | 18177 | -0.152 | -0.515 |
| Serpinc1 | 18251 | -0.158 | -0.509 |
| F2r | 18469 | -0.176 | -0.509 |
| C130027e04rik | 18535 | -0.181 | -0.501 |
| Tfpi | 18537 | -0.181 | -0.490 |
| Procr | 18691 | -0.197 | -0.486 |
| Nts | 18694 | -0.197 | -0.474 |
| Ptgs2 | 18747 | -0.203 | -0.464 |
| Fgg | 19038 | -0.248 | -0.464 |
| Vwf | 19041 | -0.248 | -0.449 |
| F2rl3 | 19045 | -0.248 | -0.434 |
| Serpine1 | 19119 | -0.262 | -0.422 |
| Agt | 19404 | -0.329 | -0.416 |
| Itgav | 19450 | -0.343 | -0.397 |
| Gja4 | 19565 | -0.390 | -0.379 |
| Irf8 | 19599 | -0.408 | -0.356 |
| Ednrb | 19728 | -0.490 | -0.333 |
| Tbx1 | 19786 | -0.535 | -0.303 |
| Tfpi2 | 19879 | -0.638 | -0.269 |
| F13a1 | 19902 | -0.681 | -0.229 |
| Serpinb2 | 19966 | -0.856 | -0.180 |
| Serpind1 | 19975 | -0.880 | -0.127 |
| Ccr1 | 20007 | -1.021 | -0.067 |
| F5 | 20014 | -1.126 | 0.001 |

RANK= position of genes in the context of the ranked list of array genes; RMS = the ranked metric score; RES = the running enrichment score.

note: negative RES indicates enrichment in zone III

**Table S3-3. Phosphatase transcripts enriched in zone I vs. III comparisons.**

| HUGO gene symbol | RANK | RMS | RES |
| --- | --- | --- | --- |
| Bpgm | 16961 | -0.090 | -0.448 |
| Ppp2cb | 16976 | -0.090 | -0.447 |
| Ppm1h | 17017 | -0.092 | -0.447 |
| Inpp5e | 17041 | -0.093 | -0.447 |
| Ppp1r10 | 17065 | -0.093 | -0.446 |
| C230090d14 | 17100 | -0.095 | -0.446 |
| Ptp4a1 | 17125 | -0.096 | -0.446 |
| Smpd2 | 17128 | -0.096 | -0.444 |
| 5430405N12RIK | 17133 | -0.096 | -0.443 |
| Ppp1ca | 17177 | -0.097 | -0.443 |
| Inpp4b | 17231 | -0.099 | -0.444 |
| 2810423O19RIK | 17255 | -0.100 | -0.443 |
| Bc005764 | 17265 | -0.100 | -0.442 |
| D3bwg0562e | 17284 | -0.101 | -0.441 |
| Ppm1a | 17289 | -0.101 | -0.439 |
| Birc1e | 17343 | -0.104 | -0.440 |
| Ptprd | 17360 | -0.105 | -0.439 |
| C130078N17RIK | 17385 | -0.106 | -0.439 |
| Plekhe1 | 17386 | -0.106 | -0.437 |
| Cdc14b | 17422 | -0.108 | -0.436 |
| Aw742319 | 17428 | -0.108 | -0.435 |
| Hdhd3 | 17468 | -0.111 | -0.435 |
| 4932443D16RIK | 17524 | -0.114 | -0.435 |
| Aw555814 | 17545 | -0.115 | -0.434 |
| Ppp1r14c | 17547 | -0.115 | -0.432 |
| Atp6v0a2 | 17570 | -0.116 | -0.431 |
| Pign | 17572 | -0.116 | -0.429 |
| Pon2 | 17591 | -0.116 | -0.428 |
| Tep1 | 17643 | -0.119 | -0.429 |
| Ppp2r2a | 17644 | -0.119 | -0.426 |
| Atp9a | 17672 | -0.121 | -0.426 |
| Pld2 | 17675 | -0.121 | -0.423 |
| E430028B21RIK | 17683 | -0.121 | -0.422 |
| Ppm1f | 17718 | -0.123 | -0.421 |
| 9130017A15RIK | 17747 | -0.124 | -0.420 |
| Frap1 | 17780 | -0.126 | -0.420 |
| Ppp2r5e | 17883 | -0.132 | -0.422 |
| Entpd1 | 17936 | -0.134 | -0.423 |
| Ptpro | 17942 | -0.135 | -0.420 |
| D6wsu116e | 17965 | -0.137 | -0.419 |
| Ppp1r14b | 17969 | -0.137 | -0.417 |
| Dusp7 | 17980 | -0.138 | -0.415 |

**Table S3-4. Phosphatase transcripts enriched in zone I vs. III comparisons.**

| HUGO gene symbol | RANK | RMS | RES |
| --- | --- | --- | --- |
| B430315C20RIK | 18002 | -0.139 | -0.413 |
| Cyba | 18003 | -0.139 | -0.411 |
| Ppap2a | 18098 | -0.146 | -0.413 |
| Ppp2r1b | 18121 | -0.148 | -0.411 |
| Dusp9 | 18130 | -0.148 | -0.409 |
| Enpp4 | 18220 | -0.156 | -0.411 |
| Inpp5b | 18266 | -0.159 | -0.410 |
| Ptprs | 18272 | -0.159 | -0.408 |
| C130085G02RIK | 18323 | -0.164 | -0.407 |
| Bc028801 | 18366 | -0.167 | -0.406 |
| Irf6 | 18373 | -0.168 | -0.404 |
| 2310028N02RIK | 18379 | -0.169 | -0.401 |
| Herc1 | 18405 | -0.171 | -0.399 |
| D830019K17RIK | 18417 | -0.172 | -0.396 |
| Atp1a1 | 18435 | -0.173 | -0.394 |
| Ccrn4l | 18506 | -0.179 | -0.394 |
| Nudt9 | 18532 | -0.181 | -0.392 |
| Ptpn6 | 18583 | -0.186 | -0.392 |
| Ptprg | 18601 | -0.188 | -0.389 |
| Ptprm | 18657 | -0.194 | -0.388 |
| Ptprr | 18665 | -0.195 | -0.385 |
| Ssh1 | 18676 | -0.195 | -0.382 |
| Ppp2r5a | 18728 | -0.201 | -0.381 |
| Pgam2 | 18746 | -0.203 | -0.378 |
| Ptpn18 | 18773 | -0.206 | -0.376 |
| Enpp1 | 18818 | -0.212 | -0.374 |
| Dusp3 | 18820 | -0.213 | -0.370 |
| Ptprb | 18842 | -0.217 | -0.367 |
| Nudt4 | 18851 | -0.218 | -0.364 |
| Ppm1j | 18866 | -0.219 | -0.361 |
| Enpp3 | 18873 | -0.220 | -0.357 |
| Dusp2 | 18890 | -0.223 | -0.354 |
| Ppp1r13b | 18895 | -0.224 | -0.350 |
| Atp2c1 | 18896 | -0.224 | -0.346 |
| Synj1 | 18914 | -0.226 | -0.343 |
| Pten | 18946 | -0.232 | -0.340 |
| D8wsu151e | 19013 | -0.244 | -0.339 |
| Dusp1 | 19019 | -0.244 | -0.335 |
| Birc1b | 19049 | -0.249 | -0.332 |
| Ptpn12 | 19051 | -0.250 | -0.327 |
| Ptprf | 19073 | -0.253 | -0.324 |
| B430203m17rik | 19127 | -0.263 | -0.322 |

**Table S3-5. Phosphatase transcripts enriched in zone I vs. III comparisons.**

| HUGO gene symbol | RANK | RMS | RES |
| --- | --- | --- | --- |
| Ssh2 | 19138 | -0.264 | -0.317 |
| Inpp5a | 19202 | -0.275 | -0.315 |
| Bc036718 | 19210 | -0.277 | -0.311 |
| Inpp5d | 19225 | -0.280 | -0.306 |
| Ptplb | 19237 | -0.283 | -0.302 |
| Ptpn1 | 19286 | -0.295 | -0.299 |
| Cmya4 | 19433 | -0.338 | -0.300 |
| Frmd3 | 19459 | -0.346 | -0.295 |
| Nav1 | 19464 | -0.348 | -0.289 |
| Dusp6 | 19536 | -0.376 | -0.286 |
| Kiaa1274 | 19552 | -0.384 | -0.280 |
| Grb14 | 19742 | -0.497 | -0.280 |
| Atp10d | 19819 | -0.572 | -0.274 |
| Ptp4a3 | 19820 | -0.572 | -0.263 |
| Ptprv | 19825 | -0.575 | -0.253 |
| Smpd3 | 19829 | -0.579 | -0.243 |
| Ptpre | 19871 | -0.632 | -0.233 |
| Bmp2k | 19883 | -0.650 | -0.222 |
| Ptpn13 | 19896 | -0.670 | -0.211 |
| Tcirg1 | 19898 | -0.678 | -0.198 |
| Ppfibp2 | 19903 | -0.683 | -0.186 |
| Enpp6 | 19930 | -0.748 | -0.174 |
| Ptpn22 | 19936 | -0.761 | -0.160 |
| Entpd3 | 19937 | -0.762 | -0.146 |
| Pstpip1 | 19941 | -0.774 | -0.133 |
| Akp2 | 19949 | -0.801 | -0.118 |
| 5133401H06RIK | 19951 | -0.806 | -0.104 |
| Ptprz1 | 19964 | -0.844 | -0.089 |
| 4933405A16RIK | 19986 | -0.933 | -0.073 |
| Ptprc | 19996 | -0.982 | -0.056 |
| Ppm1e | 19998 | -0.987 | -0.038 |
| Acp5 | 20011 | -1.061 | -0.019 |
| Dusp4 | 20017 | -1.142 | 0.001 |

RANK= position of genes in the context of the ranked list of array genes

RMS = the ranked metric score

RES = the running enrichment score

note: negative RES indicates enrichment in zone III

**Table S3-6. Cartilage transcripts enriched in zones I vs. III comparisons.**

| HUGO gene symbol | RANK | RMS | RES |
| --- | --- | --- | --- |
| Csk | 16712 | -0.082 | -0.445 |
| Mapk4 | 16728 | -0.082 | -0.444 |
| Tubb3 | 16742 | -0.083 | -0.443 |
| H2-Eb1 | 16758 | -0.083 | -0.442 |
| Mapk9 | 16766 | -0.084 | -0.440 |
| Mapk6 | 16767 | -0.084 | -0.439 |
| Gna12 | 16795 | -0.084 | -0.438 |
| Smarca5 | 16848 | -0.086 | -0.439 |
| Brap | 16850 | -0.086 | -0.437 |
| Fos | 16877 | -0.087 | -0.436 |
| Smad7 | 16892 | -0.088 | -0.435 |
| Mapkapk2 | 16992 | -0.091 | -0.438 |
| Diaph1 | 16999 | -0.091 | -0.437 |
| Dock4 | 17025 | -0.092 | -0.436 |
| Rhoa | 17071 | -0.094 | -0.436 |
| 6430596G11RIK | 17137 | -0.096 | -0.437 |
| Rnasel | 17139 | -0.096 | -0.435 |
| Vim | 17163 | -0.097 | -0.434 |
| Arsa | 17201 | -0.098 | -0.434 |
| Il27ra | 17291 | -0.101 | -0.436 |
| Tnfrsf19 | 17348 | -0.104 | -0.437 |
| Jak3 | 17467 | -0.111 | -0.441 |
| Phf21a | 17482 | -0.111 | -0.439 |
| Fosb | 17531 | -0.114 | -0.439 |
| D130043N08RIK | 17536 | -0.114 | -0.436 |
| Rhog | 17544 | -0.115 | -0.434 |
| Ctgf | 17602 | -0.117 | -0.435 |
| Creb1 | 17622 | -0.118 | -0.433 |
| Spred2 | 17637 | -0.119 | -0.431 |
| E430027O22RIK | 17680 | -0.121 | -0.431 |
| Ngfb | 17797 | -0.127 | -0.434 |
| Stat3 | 17849 | -0.130 | -0.434 |
| Ntf5 | 17858 | -0.130 | -0.431 |
| Map2k3 | 17881 | -0.132 | -0.429 |
| Srf | 17884 | -0.132 | -0.427 |
| Smurf1 | 17990 | -0.138 | -0.429 |
| Rassf7 | 18016 | -0.140 | -0.427 |
| Ceacam2 | 18036 | -0.142 | -0.425 |
| Bcl6 | 18043 | -0.143 | -0.422 |
| Ep300 | 18071 | -0.144 | -0.420 |
| Socs2 | 18164 | -0.151 | -0.422 |
| Chuk | 18348 | -0.166 | -0.427 |

**Table S3-7. Cartilage transcripts enriched in zones I vs. III comparisons.**

| HUGO gene symbol | RANK | RMS | RES |
| --- | --- | --- | --- |
| Map2k2 | 18396 | -0.170 | -0.426 |
| Map2k5 | 18415 | -0.172 | -0.423 |
| Aa536749 | 18437 | -0.173 | -0.421 |
| Spry1 | 18491 | -0.178 | -0.419 |

**Table S3-8. MAPK transcripts enriched in zone I vs. III comparisons.**

|  |  |  |  |
| --- | --- | --- | --- |
| HUGO gene symbol | RANK | RMS | RES |
| Il1r1 | 16312 | -0.070 | -0.440 |
| Casp3 | 16317 | -0.071 | -0.438 |
| Hspb1 | 16320 | -0.071 | -0.435 |
| Map2k7 | 16339 | -0.071 | -0.434 |
| Ppp3r1 | 16409 | -0.073 | -0.435 |
| Pla2g2f | 16416 | -0.073 | -0.433 |
| Tgfbr2 | 16514 | -0.075 | -0.435 |
| Rras2 | 16574 | -0.077 | -0.436 |
| 2610021I23RIK | 16632 | -0.079 | -0.436 |
| Prkaca | 16693 | -0.081 | -0.437 |
| Mapk4 | 16728 | -0.082 | -0.436 |
| Ppm1b | 16741 | -0.083 | -0.434 |
| Mapk9 | 16766 | -0.084 | -0.432 |
| Mapk6 | 16767 | -0.084 | -0.429 |
| Dok2 | 16843 | -0.086 | -0.430 |
| Brap | 16850 | -0.086 | -0.428 |
| Mapkapk2 | 16992 | -0.091 | -0.432 |
| Mapk3 | 17056 | -0.093 | -0.432 |
| Map2k1 | 17077 | -0.094 | -0.430 |
| Map3k6 | 17092 | -0.094 | -0.428 |
| Rasgrp2 | 17239 | -0.100 | -0.432 |
| Ppm1a | 17289 | -0.101 | -0.431 |
| Rps6ka1 | 17296 | -0.102 | -0.428 |
| Nras | 17319 | -0.103 | -0.426 |
| Mapk14 | 17435 | -0.109 | -0.428 |
| Fgfr1 | 17554 | -0.115 | -0.430 |
| Pla2g12a | 17581 | -0.116 | -0.428 |
| Spred2 | 17637 | -0.119 | -0.427 |
| Fgf13 | 17650 | -0.120 | -0.423 |
| Nfkb1 | 17719 | -0.123 | -0.423 |
| Ikbkb | 17728 | -0.123 | -0.419 |
| Tnfrsf1a | 17742 | -0.124 | -0.416 |
| Casp8 | 17746 | -0.124 | -0.412 |
| Ngfb | 17797 | -0.127 | -0.410 |
| Pla2g6 | 17806 | -0.128 | -0.407 |
| Map2k3 | 17881 | -0.132 | -0.406 |
| Evi1 | 17887 | -0.132 | -0.402 |
| Dusp7 | 17980 | -0.138 | -0.402 |
| Casp7 | 18015 | -0.140 | -0.399 |
| Rassf7 | 18016 | -0.140 | -0.395 |
| Dusp9 | 18130 | -0.148 | -0.396 |
| Mknk2 | 18235 | -0.156 | -0.396 |

**Table S3-9. MAPK transcripts enriched in zone I vs. III comparisons.**

|  |  |  |  |
| --- | --- | --- | --- |
| HUGO gene symbol | RANK | RMS | RES |
| Chuk | 18348 | -0.166 | -0.396 |
| Map2k5 | 18415 | -0.172 | -0.394 |
| Gadd45b | 18445 | -0.174 | -0.390 |
| Mapk11 | 18516 | -0.179 | -0.387 |
| Mapk12 | 18530 | -0.181 | -0.382 |
| 1500003O03RIK | 18573 | -0.185 | -0.378 |
| Map3k8 | 18575 | -0.185 | -0.372 |
| Cacnb3 | 18651 | -0.193 | -0.370 |
| Kras2 | 18654 | -0.193 | -0.364 |
| Ptprr | 18665 | -0.195 | -0.358 |
| Ssh1 | 18676 | -0.195 | -0.352 |
| Map3k5 | 18779 | -0.207 | -0.350 |
| Casp6 | 18798 | -0.209 | -0.344 |
| Dusp3 | 18820 | -0.213 | -0.339 |
| Prkx | 18869 | -0.220 | -0.334 |
| Dusp2 | 18890 | -0.223 | -0.328 |
| B230120H23RIK | 18897 | -0.224 | -0.321 |
| Efna1 | 18901 | -0.225 | -0.314 |
| Gab1 | 18924 | -0.228 | -0.307 |
| Prkcb1 | 18929 | -0.229 | -0.300 |
| Arrb2 | 18967 | -0.235 | -0.294 |
| Sipa1 | 18983 | -0.238 | -0.287 |
| Dusp1 | 19019 | -0.244 | -0.281 |
| Pla2g10 | 19053 | -0.250 | -0.275 |
| Rasa3 | 19068 | -0.252 | -0.267 |
| Ssh2 | 19138 | -0.264 | -0.262 |
| Nlk | 19194 | -0.273 | -0.256 |
| Fgf3 | 19303 | -0.299 | -0.252 |
| Casp4 | 19319 | -0.303 | -0.242 |
| Cd14 | 19353 | -0.312 | -0.234 |
| Rasgrp1 | 19386 | -0.324 | -0.225 |
| Raf1 | 19478 | -0.353 | -0.218 |
| Pla2g4a | 19498 | -0.362 | -0.207 |
| Mbp | 19512 | -0.368 | -0.196 |
| Dusp6 | 19536 | -0.376 | -0.185 |
| Pdgfb | 19555 | -0.385 | -0.173 |
| Bdnf | 19591 | -0.405 | -0.162 |
| Map4k4 | 19638 | -0.431 | -0.150 |
| Tgfb1 | 19752 | -0.504 | -0.139 |
| Fgf7 | 19760 | -0.511 | -0.123 |
| Casp1 | 19780 | -0.528 | -0.107 |
| Gadd45a | 19810 | -0.563 | -0.090 |

**Table S3-10. MAPK transcripts enriched in zone I vs. III comparisons.**

|  |  |  |  |
| --- | --- | --- | --- |
| HUGO gene symbol | RANK | RMS | RES |
| Pdgfrb | 19864 | -0.613 | -0.073 |
| Rac2 | 19876 | -0.636 | -0.053 |
| Gadd45g | 19923 | -0.732 | -0.031 |
| Dusp4 | 20017 | -1.142 | 0.001 |

RANK= position of genes in the context of the ranked list of array genes

RMS = the ranked metric score

RES = the running enrichment score

note: negative RES indicates enrichment in zone III

**Table S3-11. Cartilage transcripts enriched in zone I vs. III comparisons.**

| HUGO gene symbol | RANK | RMS | RES |
| --- | --- | --- | --- |
| Sox9 | 114 | 0.606 | 0.084 |
| Otor | 135 | 0.579 | 0.169 |
| Lect1 | 144 | 0.564 | 0.253 |
| Mia1 | 265 | 0.438 | 0.312 |
| Chi3l1 | 273 | 0.433 | 0.376 |
| Bmpr1b | 279 | 0.428 | 0.439 |
| Matn1 | 473 | 0.330 | 0.478 |
| Hapln1 | 860 | 0.235 | 0.494 |
| Agc1 | 1151 | 0.195 | 0.508 |
| Cilp | 1256 | 0.184 | 0.531 |
| Thra | 1362 | 0.175 | 0.551 |

RANK= position of genes in the context of the ranked list of array genes

RMS = the ranked metric score

RES = the running enrichment score

note: positive RES indicates enrichment in zone I

**Table S3-12. Bone transcripts enriched in zone I vs. III comparisons.**

| HUGO gene symbol | RANK | RMS | RES |
| --- | --- | --- | --- |
| Bmp7 | 18835 | -0.215 | -0.481 |
| Nox4 | 19017 | -0.244 | -0.479 |
| Bmp1 | 19072 | -0.253 | -0.470 |
| Mvp | 19190 | -0.273 | -0.463 |
| Clecsf9 | 19277 | -0.293 | -0.454 |
| Bmp2 | 19452 | -0.343 | -0.447 |
| Fn1 | 19458 | -0.346 | -0.432 |
| Mbp | 19512 | -0.368 | -0.418 |
| 9830115L13RIK | 19588 | -0.403 | -0.403 |
| Slc16a10 | 19630 | -0.426 | -0.386 |
| Cpeb4 | 19668 | -0.448 | -0.367 |
| Pthr1 | 19767 | -0.516 | -0.348 |
| Schip1 | 19768 | -0.517 | -0.325 |
| Cd38 | 19823 | -0.573 | -0.301 |
| Mmrn1 | 19866 | -0.618 | -0.275 |
| Bmp2k | 19883 | -0.650 | -0.247 |
| Mepe | 19940 | -0.772 | -0.214 |
| Akp2 | 19949 | -0.801 | -0.178 |
| Bmp8a | 19985 | -0.920 | -0.138 |
| Ibsp | 20036 | -1.447 | -0.074 |
| Tnfsf11 | 20039 | -1.641 | 0.000 |

RANK= position of genes in the context of the ranked list of array genes

RMS = the ranked metric score

RES = the running enrichment score

note: negative RES indicates enrichment in zone III
