# Supplementary material for: Genome-Wide Analyses of Gene Expression during Mouse Endochondral Ossification
Source: PLoS One. 2010 Jan 13;5(1):e8693. doi: 10.1371/journal.pone.0008693 (PMC2805713; doi:10.1371/journal.pone.0008693)
Supplement: Table S4 — GSEA enrichment of micromass culture data using c1 gene sets. (0.15 MB DOC) [file pone.0008693.s004.doc]

**Table S4-1.** **GSEA enrichment of micromass culture data using c1 gene sets.**

3 vs. 9/I vs. II

| Number | Gene Set Name | SIZE | ES | NES* | NOM p-val | FDR q-val |
| --- | --- | --- | --- | --- | --- | --- |
| 1 | CHRXQ26 | 16 | 0.690 | 1.750 | 0.007 | 0.502 |
| 2# | CHR14Q21 | 17 | 0.652 | 1.700 | 0.019 | 0.396 |
| 3 | CHR2P22 | 18 | 0.488 | 1.288 | 0.168 | 0.814 |
| 4 | CHR11Q22 | 21 | -0.725 | -1.880 | 0.001 | 0.044 |
| 5 | CHR15Q26 | 28 | -0.513 | -1.412 | 0.077 | 0.609 |
| 6 | CHR2Q24 | 28 | -0.498 | -1.368 | 0.096 | 0.598 |

* Negative values indicate correlation with day 9 of micromass culture

# Gene sets that exhibit opposite enrichment patterns between MM and MD

**Table S4-2. GSEA enrichment of microdissected growth plate data using c1 gene sets.**

3 vs. 9/I vs. II

| Number | Gene Set Name | SIZE | ES | NES* | NOM p-val | FDR q-val |
| --- | --- | --- | --- | --- | --- | --- |
| 1 | CHRXQ26 | 22 | 0.637 | 1.609 | 0.023 | 0.208 |
| 2# | CHR14Q21 | 28 | -0.522 | -1.532 | 0.032 | 0.373 |
| 3 | CHR2P22 | 23 | 0.554 | 1.424 | 0.075 | 0.498 |
| 4 | CHR11Q22 | 37 | -0.398 | -1.235 | 0.169 | 0.711 |
| 5 | CHR15Q26 | 36 | -0.558 | -1.726 | 0.007 | 0.176 |
| 6 | CHR2Q24 | 32 | -0.459 | -1.384 | 0.093 | 0.544 |

* Negative values indicate correlation with zone II

# Gene sets that exhibit opposite enrichment patterns between MM and MD

**Table S4-3. GSEA enrichment of micromass culture data using c1 gene sets.**

9 vs. 15/II vs. III

| Number | Gene Set Name | SIZE | ES | NES* | NOM p-val | FDR q-val |
| --- | --- | --- | --- | --- | --- | --- |
| 1 | CHRXQ23 | 15 | 0.646 | 1.645 | 0.021 | 0.460 |
| 2 | CHR12Q21 | 19 | 0.570 | 1.489 | 0.051 | 0.675 |
| 3 | CHR11Q22 | 21 | -0.711 | -1.821 | 0.002 | 0.146 |
| 4 | CHR1Q23 | 67 | -0.536 | -1.726 | 0.001 | 0.164 |
| 5# | CHR14Q22 | 20 | -0.613 | -1.529 | 0.046 | 0.452 |
| 6 | CHR16P11 | 42 | -0.513 | -1.501 | 0.029 | 0.494 |
| 7 | CHR1Q31 | 21 | -0.572 | -1.453 | 0.085 | 0.556 |
| 8 | CHR15Q21 | 39 | -0.481 | -1.394 | 0.077 | 0.684 |
| 9 | CHR10 | 155 | -0.375 | -1.350 | 0.036 | 0.633 |
| 10 | CHR8Q21 | 30 | -0.483 | -1.319 | 0.127 | 0.672 |

* Negative values indicate correlation with day 15 of micromass culture

# Gene sets that exhibit opposite enrichment patterns between MM and MD

**Table S4-4. GSEA enrichment of microdissected growth plate data using c1 gene sets.**

9 vs. 15/II vs. III

| Number | Gene Set Name | SIZE | ES | NES* | NOM p-val | FDR q-val |
| --- | --- | --- | --- | --- | --- | --- |
| 1 | CHR12Q21 | 27 | 0.405 | 1.205 | 0.203 | 0.515 |
| 2 | CHRXQ23 | 17 | 0.489 | 1.286 | 0.181 | 0.398 |
| 3 | CHR11Q22 | 28 | -0.593 | -1.555 | 0.025 | 0.286 |
| 4 | CHR1Q23 | 68 | -0.495 | -1.509 | 0.016 | 0.282 |
| 5# | CHR14Q22 | 24 | 0.574 | 1.662 | 0.015 | 0.105 |
| 6 | CHR16P11 | 45 | -0.557 | -1.603 | 0.012 | 0.217 |
| 7 | CHR1Q31 | 21 | -0.807 | -2.004 | 0.000 | 0.002 |
| 8 | CHR15Q21 | 42 | -0.579 | -1.631 | 0.007 | 0.209 |
| 9 | CHR10 | 161 | -0.465 | -1.573 | 0.002 | 0.264 |
| 10 | CHR8Q21 | 32 | -0.653 | -1.743 | 0.003 | 0.177 |

* Negative values indicate correlation with zone III

# Gene sets that exhibit opposite enrichment patterns between MM and MD

**Table S4-5. GSEA enrichment of micromass culture data using c1 gene sets.**

3 vs. 15/I vs. III

| Number | Gene Set Name | SIZE | ES | NES* | NOM p-val | FDR q-val |
| --- | --- | --- | --- | --- | --- | --- |
| 1 | CHRXQ26 | 16 | 0.666 | 1.629 | 0.023 | 1.000 |
| 2# | CHR16Q24 | 32 | 0.558 | 1.602 | 0.014 | 0.704 |
| 3 | CHR14Q21 | 17 | 0.592 | 1.489 | 0.061 | 0.672 |
| 4 | CHR4Q25 | 15 | 0.587 | 1.408 | 0.096 | 0.728 |
| 5# | CHR10Q21 | 19 | 0.542 | 1.380 | 0.096 | 0.630 |
| 6 | CHR11Q22 | 21 | -0.749 | -1.866 | 0.000 | 0.036 |
| 7 | CHR14Q22 | 20 | -0.751 | -1.860 | 0.001 | 0.021 |
| 8 | CHR4Q22 | 18 | -0.658 | -1.572 | 0.030 | 0.448 |
| 9 | CHR1Q31 | 21 | -0.628 | -1.557 | 0.031 | 0.413 |
| 10 | CHR1P22 | 25 | -0.603 | -1.534 | 0.029 | 0.359 |
| 11 | CHR11Q12 | 56 | -0.472 | -1.412 | 0.036 | 0.635 |
| 12 | CHR6Q23 | 26 | -0.545 | -1.394 | 0.079 | 0.515 |
| 13 | CHR16Q12 | 22 | -0.537 | -1.330 | 0.114 | 0.568 |

* Negative values indicate correlation with day 15 of micromass culture

# Gene sets that exhibit opposite enrichment patterns between MM and MD

**Table S4-6. GSEA enrichment of microdissected growth plate data using c1 gene sets.**

3 vs. 15/I vs. III

| Number | Gene Set Name | SIZE | ES | NES* | NOM p-val | FDR q-val |
| --- | --- | --- | --- | --- | --- | --- |
| 1 | CHRXQ26 | 20 | 0.692 | 1.710 | 0.007 | 0.120 |
| 2# | CHR16Q24 | 36 | -0.615 | -1.607 | 0.011 | 0.345 |
| 3 | CHR14Q21 | 21 | 0.574 | 1.428 | 0.061 | 0.455 |
| 4 | CHR4Q25 | 18 | 0.683 | 1.642 | 0.013 | 0.174 |
| 5# | CHR10Q21 | 25 | -0.592 | -1.454 | 0.059 | 0.471 |
| 6 | CHR11Q22 | 28 | -0.655 | -1.646 | 0.011 | 0.283 |
| 7 | CHR14Q22 | 24 | 0.483 | 1.232 | 0.197 | 0.676 |
| 8 | CHR4Q22 | 22 | -0.733 | -1.744 | 0.004 | 0.162 |
| 9 | CHR1Q31 | 21 | -0.726 | -1.748 | 0.004 | 0.309 |
| 10 | CHR1P22 | 27 | -0.579 | -1.435 | 0.069 | 0.448 |
| 11 | CHR11Q12 | 59 | -0.492 | -1.412 | 0.042 | 0.474 |
| 12 | CHR6Q23 | 27 | -0.632 | -1.579 | 0.020 | 0.326 |
| 13 | CHR16Q12 | 26 | -0.628 | -1.549 | 0.030 | 0.302 |

* Negative values indicate correlation with zone III

# Gene sets that exhibit opposite enrichment patterns between MM and MD
