# Supplementary material for: Genome-Wide Analyses of Gene Expression during Mouse Endochondral Ossification
Source: PLoS One. 2010 Jan 13;5(1):e8693. doi: 10.1371/journal.pone.0008693 (PMC2805713; doi:10.1371/journal.pone.0008693)
Supplement: Table S5 — GSEA enrichment of micromass culture data using c2 gene sets. (0.13 MB DOC) [file pone.0008693.s005.doc]

**Table S5-1. GSEA enrichment of micromass culture data using c2 gene sets.**

3 vs. 9/I vs. II

| Number | Gene Set Name | SIZE | ES | NES* | NOM p-val | FDR q-val |
| --- | --- | --- | --- | --- | --- | --- |
| 1 | PROTEASOMEPATHWAY | 21 | 0.709 | 1.914 | 0.001 | 0.013 |
| 2 | ELECTRON TRANSPORT CHAIN | 79 | 0.451 | 1.602 | 0.013 | 0.146 |
| 3 | VOXPHOS | 73 | 0.439 | 1.524 | 0.014 | 0.2 |
| 4# | MATRIX METALLOPROTEINASES | 22 | -0.69 | -1.788 | 0.004 | 0.065 |

* Negative values indicate correlation with day 9 of micromass culture

# Gene sets that exhibit the same enrichment patterns between MM and MD

**Table S5-2. GSEA enrichment of microdissected growth plate data using c2 gene sets.**

3 vs. 9/I vs. II

| Number | Gene Set Name | SIZE | ES | NES* | NOM p-val | FDR q-val |
| --- | --- | --- | --- | --- | --- | --- |
| 1 | VOXPHOS | 74 | -0.555 | -1.999 | 0 | 0.043 |
| 2 | ELECTRON TRANSPORT CHAIN | 88 | -0.494 | -1.823 | 0.001 | 0.148 |
| 3 | PROTEASOMEPATHWAY | 21 | -0.633 | -1.738 | 0.011 | 0.162 |
| 4# | MATRIX METALLOPROTEINASES | 23 | -0.566 | -1.583 | 0.031 | 0.21 |

* Negative values indicate correlation with zone II

# Gene sets that exhibit the same enrichment patterns between MM and MD

**Table S5-3. GSEA enrichment of micromass culture data using c2 gene sets.**

9 vs. 15/II vs. III

| Number | Gene Set Name | SIZE | ES | NES* | NOM p-val | FDR q-val |
| --- | --- | --- | --- | --- | --- | --- |
| 1 | PROTEASOMEPATHWAY | 21 | 0.494 | 1.368 | 0.107 | 0.562 |
| 2 | SIG BCR SIGNALING PATHWAY | 44 | -0.649 | -1.946 | 0 | 0.009 |
| 3 | NKTPATHWAY | 28 | -0.706 | -1.889 | 0.001 | 0.018 |
| 4 | SIG PIP3 SIGNALING IN B CELLS | 32 | -0.667 | -1.871 | 0.002 | 0.021 |
| 5 | FCER1PATHWAY | 37 | -0.638 | -1.831 | 0.001 | 0.029 |
| 6 | TOLLPATHWAY | 33 | -0.641 | -1.797 | 0.005 | 0.036 |
| 7 | NKCELLSPATHWAY | 18 | -0.729 | -1.785 | 0.005 | 0.039 |
| 8 | CELL SURFACE RECEPTOR | 110 | -0.517 | -1.779 | 0 | 0.04 |
| 9 | EMT UP | 56 | -0.568 | -1.758 | 0.002 | 0.047 |
| 10 | ST B CELL ANTIGEN RECEPTOR | 35 | -0.61 | -1.719 | 0.007 | 0.063 |
| 11 | CELL ADHESION | 153 | -0.467 | -1.675 | 0 | 0.092 |
| 12 | ST T CELL SIGNAL TRANSDUCTION | 38 | -0.562 | -1.618 | 0.014 | 0.112 |
| 13 | IL6PATHWAY | 20 | -0.631 | -1.587 | 0.027 | 0.12 |
| 14# | FATTY ACID METABOLISM | 22 | -0.582 | -1.506 | 0.051 | 0.193 |
| 15 | GO ROS | 25 | -0.568 | -1.505 | 0.047 | 0.189 |
| 16 | ETSPATHWAY | 17 | -0.615 | -1.492 | 0.067 | 0.198 |
| 17# | CYTOKINEPATHWAY | 20 | -0.579 | -1.454 | 0.077 | 0.241 |
| 18 | MAPKPATHWAY | 78 | -0.443 | -1.446 | 0.034 | 0.242 |

* Negative values indicate correlation with day 15 of micromass culture

# Gene sets that exhibit the same enrichment patterns between MM and MD

**Table S5-4. GSEA enrichment of microdissected growth plate data using c2 gene sets.**

9 vs. 15/II vs. III

| Number | Gene Set Name | SIZE | ES | NES* | NOM p-val | FDR q-val |
| --- | --- | --- | --- | --- | --- | --- |
| 1 | PROTEASOMEPATHWAY | 21 | -0.517 | -1.463 | 0.057 | 0.431 |
| 2 | SIG BCR SIGNALING PATHWAY | 43 | 0.607 | 1.707 | 0.002 | 0.019 |
| 3 | NKTPATHWAY | 27 | 0.715 | 1.846 | 0.001 | 0.004 |
| 4 | SIG PIP3 SIGNALING IN B CELLS | 31 | 0.637 | 1.73 | 0.003 | 0.016 |
| 5 | FCER1PATHWAY | 35 | 0.66 | 1.78 | 0.001 | 0.009 |
| 6 | TOLLPATHWAY | 34 | 0.674 | 1.828 | 0 | 0.005 |
| 7 | NKCELLSPATHWAY | 17 | 0.712 | 1.692 | 0.007 | 0.021 |
| 8 | CELL SURFACE RECEPTOR | 117 | 0.564 | 1.843 | 0 | 0.004 |
| 9 | EMT UP | 45 | 0.669 | 1.922 | 0 | 0.001 |
| 10 | ST B CELL ANTIGEN RECEPTOR | 34 | 0.615 | 1.673 | 0.008 | 0.023 |
| 11 | CELL ADHESION | 160 | 0.521 | 1.758 | 0 | 0.011 |
| 12 | ST T CELL SIGNAL TRANSDUCTION | 43 | 0.676 | 1.915 | 0 | 0.001 |
| 13 | IL6PATHWAY | 20 | 0.722 | 1.766 | 0.003 | 0.011 |
| 14# | FATTY ACID METABOLISM | 61 | -0.378 | -1.37 | 0.053 | 0.434 |
| 15 | GO ROS | 25 | 0.677 | 1.725 | 0.004 | 0.016 |
| 16 | ETSPATHWAY | 17 | 0.787 | 1.867 | 0 | 0.003 |
| 17# | CYTOKINEPATHWAY | 20 | -0.487 | -1.352 | 0.117 | 0.456 |
| 18 | MAPKPATHWAY | 81 | 0.536 | 1.684 | 0.001 | 0.022 |

* Negative values indicate correlation with zone III

# Gene sets that exhibit the same enrichment patterns between MM and MD

**Table S5-5. GSEA enrichment of micromass culture data using c2 gene sets.**

3 vs. 15/I vs. III

| Number | Gene Set Name | SIZE | ES | NES* | NOM p-val | FDR q-val |
| --- | --- | --- | --- | --- | --- | --- |
| 1 | NKTPATHWAY | 28 | -0.695 | -1.825 | 0.001 | 0.019 |
| 2 | MATRIX METALLOPROTEINASES | 22 | -0.667 | -1.667 | 0.010 | 0.070 |
| 3 | GO ROS | 25 | -0.664 | -1.705 | 0.006 | 0.063 |

*Negative values indicate correlation with day 15 of micromass culture

**Table S5-6. GSEA enrichment of microdissected growth plate data using c2 gene sets.**

3 vs. 15/I vs. III

| Number | Gene Set Name | SIZE | ES | NES* | NOM p-val | FDR q-val |
| --- | --- | --- | --- | --- | --- | --- |
| 1 | NKTPATHWAY | 27 | -0.785 | -1.973 | 0.000 | 0.002 |
| 2 | MATRIX METALLOPROTEINASES | 23 | -0.780 | -1.886 | 0.001 | 0.007 |
| 3 | GO ROS | 25 | -0.732 | -1.802 | 0.002 | 0.019 |

* Negative values indicate correlation with zone III
