# Supplementary material for: Genome-Wide Analyses of Gene Expression during Mouse Endochondral Ossification
Source: PLoS One. 2010 Jan 13;5(1):e8693. doi: 10.1371/journal.pone.0008693 (PMC2805713; doi:10.1371/journal.pone.0008693)
Supplement: Table S6 — GSEA enrichment of micromass culture data using c3 gene sets. (0.27 MB DOC) [file pone.0008693.s006.doc]

**Table S6-1. GSEA enrichment of micromass culture data using c3 gene sets.**

3 vs. 9/I vs. II

| Number | Gene Set Name | SIZE | ES | NES* | NOM p-val | FDR q-val |
| --- | --- | --- | --- | --- | --- | --- |
| 1 | SGCGSSAAA | 104 | 0.598 | 2.229 | <0.001 | 0.001 |
| 2 | ACAWYAAAG | 49 | 0.567 | 1.861 | 0.001 | 0.008 |
| 3 | YWATTWNNRGCT | 40 | 0.555 | 1.725 | 0.007 | 0.026 |
| 4 | CAGNYGKNAAA | 45 | 0.544 | 1.720 | 0.008 | 0.025 |
| 5 | GKCGCNNNNNNNTGAYG | 35 | 0.545 | 1.661 | 0.010 | 0.038 |
| 6 | ACAWNRNSRCGG | 45 | 0.511 | 1.634 | 0.011 | 0.042 |
| 7 | TCCCRNNRTGC | 107 | 0.425 | 1.591 | 0.005 | 0.057 |
| 8 | CTTTAAR | 151 | 0.397 | 1.578 | 0.002 | 0.057 |
| 9 | TCCATTKW | 138 | 0.398 | 1.543 | 0.004 | 0.065 |
| 10 | GTCNYYATGR | 49 | 0.468 | 1.534 | 0.024 | 0.068 |
| 11 | WGGAATGY | 165 | 0.327 | 1.292 | 0.048 | 0.182 |
| 12 | WTTGKCTG | 147 | 0.333 | 1.290 | 0.058 | 0.181 |
| 13 | TMTCGCGANR | 93 | 0.349 | 1.275 | 0.089 | 0.186 |
| 14 | WTGAAAT | 165 | 0.322 | 1.275 | 0.044 | 0.184 |
| 15 | TTANWNANTGGM | 30 | 0.429 | 1.259 | 0.169 | 0.198 |
| 16 | GGGAGGRR | 186 | 0.306 | 1.230 | 0.069 | 0.223 |
| 17 | RGAGGAARY | 159 | -0.355 | -1.299 | 0.059 | 0.457 |
| 18 | TTGTTT | 156 | -0.334 | -1.219 | 0.118 | 0.619 |
| 19 | TGGAAA | 163 | -0.324 | -1.188 | 0.142 | 0.622 |
| 20 | CTTTGA | 154 | -0.311 | -1.129 | 0.214 | 0.741 |

* Negative values indicate correlation with day 9 of micromass culture

**Table S6-2. GSEA enrichment of microdissected growth plate data using c3 gene sets.**

3 vs. 9/I vs. II

| Number | Gene Set Name | SIZE | ES | NES* | NOM p-val | | FDR q-val |
| --- | --- | --- | --- | --- | --- | --- | --- |
| 1 | SGCGSSAAA | 112 | 0.582 | 1.924 | | <0.001 | 0.001 |
| 2 | ACAWYAAAG | 64 | 0.604 | 1.841 | | 0.001 | 0.004 |
| 3 | YWATTWNNRGCT | 42 | 0.569 | 1.620 | | 0.008 | 0.023 |
| 4 | CAGNYGKNAAA | 49 | 0.541 | 1.574 | | 0.016 | 0.034 |
| 5 | GKCGCNNNNNNNTGAYG | 37 | -0.402 | -1.267 | | 0.158 | 0.785 |
| 6 | ACAWNRNSRCGG | 48 | -0.340 | -1.113 | | 0.269 | 1.000 |
| 7 | TCCCRNNRTGC | 115 | -0.318 | -1.222 | | 0.095 | 0.705 |
| 8 | CTTTAAR | 163 | 0.511 | 1.777 | | <0.001 | 0.007 |
| 9 | TCCATTKW | 155 | 0.487 | 1.680 | | <0.001 | 0.013 |
| 10 | GTCNYYATGR | 57 | -0.311 | -1.064 | | 0.337 | 0.776 |
| 11 | WGGAATGY | 181 | 0.471 | 1.643 | | <0.001 | 0.019 |
| 12 | WTTGKCTG | 169 | 0.500 | 1.738 | | <0.001 | 0.008 |
| 13 | TMTCGCGANR | 101 | -0.205 | -0.774 | | 0.921 | 0.905 |
| 14 | WTGAAAT | 179 | 0.445 | 1.555 | | 0.002 | 0.036 |
| 15 | TTANWNANTGGM | 35 | 0.604 | 1.682 | | 0.006 | 0.013 |
| 16 | GGGAGGRR | 196 | 0.453 | 1.610 | | 0.001 | 0.025 |
| 17 | RGAGGAARY | 177 | 0.447 | 1.568 | | 0.002 | 0.033 |
| 18 | TTGTTT | 173 | 0.496 | 1.720 | | <0.001 | 0.010 |
| 19 | TGGAAA | 187 | 0.502 | 1.775 | | <0.001 | 0.007 |
| 20 | CTTTGA | 170 | 0.547 | 1.900 | | <0.001 | 0.002 |

* Negative values indicate correlation with zone II

**Table S6-3. GSEA enrichment of micromass culture data using c3 gene sets.**

9 vs. 15/II vs. III

| Number | Gene Set Name | SIZE | ES | NES* | NOM p-val | FDR q-val |
| --- | --- | --- | --- | --- | --- | --- |
| 1 | GCGSCMNTTT | 37 | 0.604 | 1.921 | 0.001 | 0.008 |
| 2 | RYTGCNNRGNAAC | 37 | 0.538 | 1.683 | 0.006 | 0.059 |
| 3 | RYAAAKNNNNNNTTGW | 51 | 0.472 | 1.567 | 0.014 | 0.083 |
| 4 | YRTCANNRCGC | 40 | 0.488 | 1.552 | 0.018 | 0.072 |
| 5 | GTTNYYNNGGTNA | 39 | 0.437 | 1.390 | 0.049 | 0.135 |
| 6 | TCCCRNNRTGC | 107 | 0.341 | 1.319 | 0.053 | 0.171 |
| 7 | GKCGCNNNNNNNTGAYG | 35 | 0.421 | 1.306 | 0.106 | 0.183 |
| 8 | RACTNNRTTTNC | 67 | 0.355 | 1.253 | 0.112 | 0.238 |
| 9 | STTTCRNTTT | 122 | -0.445 | -1.552 | 0.006 | 0.534 |
| 10 | RGAGGAARY | 159 | -0.421 | -1.515 | 0.005 | 0.383 |
| 11 | YTAAYNGCT | 94 | -0.449 | -1.512 | 0.012 | 0.262 |
| 12 | TCANNTGAY | 174 | -0.394 | -1.449 | 0.011 | 0.338 |
| 13 | GGARNTKYCCA | 46 | -0.471 | -1.396 | 0.071 | 0.418 |
| 14 | GGCKCATGS | 38 | -0.468 | -1.342 | 0.103 | 0.525 |
| 15 | GGGNNTTTCC | 89 | -0.388 | -1.296 | 0.093 | 0.622 |
| 16 | RYCACNNRNNRNCAG | 42 | -0.443 | -1.288 | 0.128 | 0.573 |
| 17 | GATAAGR | 174 | -0.339 | -1.242 | 0.092 | 0.698 |
| 18 | TGGAAA | 163 | -0.326 | -1.180 | 0.168 | 0.917 |
| 19 | YNTTTNNNANGCARM | 46 | -0.387 | -1.165 | 0.236 | 0.906 |
| 20 | RAAGNYNNCTTY | 82 | -0.345 | -1.139 | 0.247 | 0.958 |

* Negative values indicate correlation with day 15 of micromass culture

**Table S6-4. GSEA enrichment of microdissected growth plate data using c3 gene sets.**

9 vs 15/ II vs III

| Number | Gene Set Name | SIZE | ES | NES* | NOM p-val | FDR q-val |
| --- | --- | --- | --- | --- | --- | --- |
| 1 | GCGSCMNTTT | 45 | 0.276 | 0.918 | 0.595 | 0.749 |
| 2 | RYTGCNNRGNAAC | 46 | 0.224 | 0.752 | 0.876 | 0.933 |
| 3 | RYAAAKNNNNNNTTGW | 56 | 0.322 | 1.116 | 0.252 | 0.917 |
| 4 | YRTCANNRCGC | 48 | 0.275 | 0.935 | 0.584 | 0.817 |
| 5 | GTTNYYNNGGTNA | 48 | 0.295 | 1.002 | 0.453 | 0.893 |
| 6 | TCCCRNNRTGC | 115 | 0.264 | 1.038 | 0.362 | 0.979 |
| 7 | GKCGCNNNNNNNTGAYG | 37 | 0.302 | 0.961 | 0.515 | 0.877 |
| 8 | RACTNNRTTTNC | 72 | 0.392 | 1.435 | 0.015 | 0.200 |
| 9 | STTTCRNTTT | 134 | -0.572 | -1.890 | 0.000 | 0.001 |
| 10 | RGAGGAARY | 177 | -0.565 | -1.920 | 0.000 | 0.001 |
| 11 | YTAAYNGCT | 97 | -0.472 | -1.508 | 0.014 | 0.030 |
| 12 | TCANNTGAY | 185 | -0.478 | -1.640 | 0.002 | 0.009 |
| 13 | GGARNTKYCCA | 52 | -0.480 | -1.394 | 0.060 | 0.066 |
| 14 | GGCKCATGS | 43 | -0.478 | -1.342 | 0.086 | 0.098 |
| 15 | GGGNNTTTCC | 94 | -0.524 | -1.671 | 0.001 | 0.009 |
| 16 | RYCACNNRNNRNCAG | 48 | -0.484 | -1.413 | 0.058 | 0.061 |
| 17 | GATAAGR | 184 | -0.530 | -1.810 | 0.000 | 0.002 |
| 18 | TGGAAA | 187 | -0.566 | -1.918 | 0.000 | 0.001 |
| 19 | YNTTTNNNANGCARM | 48 | -0.615 | -1.788 | 0.000 | 0.003 |
| 20 | RAAGNYNNCTTY | 91 | -0.538 | -1.710 | 0.000 | 0.006 |

* Negative values indicate correlation with zone III

**Table S6-5. GSEA enrichment of micromass culture data using c3 gene sets.**

3 vs 15/I vs III

| Number | Gene Set Name | SIZE | ES | NES* | NOM p-val | FDR q-val |
| --- | --- | --- | --- | --- | --- | --- |
| 1 | SGCGSSAAA | 104 | 0.640 | 2.300 | 0.000 | 0.000 |
| 2 | GCGSCMNTTT | 37 | 0.641 | 1.891 | 0.000 | 0.003 |
| 3 | ACAWNRNSRCGG | 45 | 0.576 | 1.773 | 0.002 | 0.010 |
| 4 | YWATTWNNRGCT | 40 | 0.578 | 1.743 | 0.000 | 0.014 |
| 5 | AACWWCAANK | 81 | 0.443 | 1.514 | 0.011 | 0.054 |
| 6 | RYTAAWNNNTGAY | 40 | 0.500 | 1.514 | 0.026 | 0.052 |
| 7 | CAGGTA | 157 | 0.384 | 1.460 | 0.003 | 0.070 |
| 8 | CCAWWNAAGG | 63 | 0.433 | 1.421 | 0.040 | 0.086 |
| 9 | CCTNTMAGA | 77 | 0.409 | 1.398 | 0.036 | 0.097 |
| 10 | YATTNATC | 149 | 0.364 | 1.368 | 0.017 | 0.116 |
| 11 | STTTCRNTTT | 122 | -0.475 | -1.588 | 0.004 | 0.222 |
| 12 | RYTTCCTG | 170 | -0.417 | -1.462 | 0.005 | 0.383 |
| 13 | YTAAYNGCT | 94 | -0.451 | -1.461 | 0.019 | 0.257 |
| 14 | TGANTCA | 176 | -0.414 | -1.457 | 0.006 | 0.200 |
| 15 | RGAGGAARY | 159 | -0.420 | -1.453 | 0.009 | 0.167 |
| 16 | GGGNNTTTCC | 89 | -0.451 | -1.436 | 0.021 | 0.163 |
| 17 | GGARNTKYCCA | 46 | -0.429 | -1.251 | 0.144 | 0.615 |
| 18 | TGGAAA | 163 | -0.338 | -1.177 | 0.165 | 0.872 |
| 19 | TTCYNRGAA | 170 | -0.333 | -1.166 | 0.153 | 0.826 |
| 20 | TCANNTGAY | 174 | -0.331 | -1.153 | 0.176 | 0.808 |

* Negative values indicate correlation with day 15 of micromass culture

**Table S6-6. GSEA enrichment of microdissected growth plate data using c3 gene sets.**

3 vs 15/I vs III

| Number | Gene Set Name | SIZE | ES | NES* | NOM  p-val | FDR  q-val |
| --- | --- | --- | --- | --- | --- | --- |
| 1 | SGCGSSAAA | 112 | 0.397 | 1.351 | 0.031 | 0.366 |
| 2 | GCGSCMNTTT | 45 | 0.387 | 1.124 | 0.265 | 0.622 |
| 3 | ACAWNRNSRCGG | 48 | -0.581 | -1.611 | 0.011 | 0.106 |
| 4 | YWATTWNNRGCT | 42 | -0.530 | -1.420 | 0.057 | 0.191 |
| 5 | AACWWCAANK | 89 | 0.383 | 1.265 | 0.101 | 0.478 |
| 6 | RYTAAWNNNTGAY | 42 | 0.408 | 1.176 | 0.203 | 0.668 |
| 7 | CAGGTA | 173 | -0.395 | -1.315 | 0.043 | 0.258 |
| 8 | CCAWWNAAGG | 68 | -0.463 | -1.355 | 0.056 | 0.240 |
| 9 | CCTNTMAGA | 85 | 0.327 | 1.063 | 0.329 | 0.566 |
| 10 | YATTNATC | 178 | 0.309 | 1.113 | 0.189 | 0.571 |
| 11 | STTTCRNTTT | 134 | -0.443 | -1.440 | 0.012 | 0.168 |
| 12 | RYTTCCTG | 189 | -0.545 | -1.828 | 0.000 | 0.030 |
| 13 | YTAAYNGCT | 97 | -0.440 | -1.366 | 0.048 | 0.245 |
| 14 | TGANTCA | 200 | -0.480 | -1.623 | 0.000 | 0.116 |
| 15 | RGAGGAARY | 177 | -0.462 | -1.548 | 0.003 | 0.155 |
| 16 | GGGNNTTTCC | 94 | -0.459 | -1.417 | 0.033 | 0.188 |
| 17 | GGARNTKYCCA | 52 | -0.473 | -1.328 | 0.088 | 0.239 |
| 18 | TGGAAA | 187 | -0.422 | -1.406 | 0.013 | 0.195 |
| 19 | TTCYNRGAA | 183 | -0.402 | -1.343 | 0.026 | 0.224 |
| 20 | TCANNTGAY | 185 | -0.441 | -1.474 | 0.007 | 0.144 |

* Negative values indicate correlation with zone III
